# Exercise-Based Strategies from Warm-Up to Training: A Systematic Review of Performance Enhancement and Injury Prevention

**DOI:** 10.3390/sports14050187

**Published:** 2026-05-06

**Authors:** Wiktor Kłobuchowski, Maciej Skorulski, Kajetan Ornowski, Robert Roczniok, Adam Maszczyk, Bianca Callegari, Givago Silva Souza, Przemysław Pietraszewski, Szymon Kuliś

**Affiliations:** 1 Doctoral School, Józef Piłsudski University of Physical Education in Warsaw, 00-968 Warsaw, Poland; ms694@stud.awf.edu.pl; 2 Institute of Sport Sciences, Academy of Physical Education, 40-065 Katowice, Poland; ornowskikajtek@gmail.com (K.O.); r.roczniok@awf.katowice.pl (R.R.); a.maszczyk@awf.katowice.pl (A.M.); p.pietraszewski@awf.katowice.pl (P.P.); 3 Instituto de Ciências da Saúde, Universidade Federal do Pará, Belém 66075-110, Brazil; callegaribi@gmail.com; 4 Instituto de Ciências Biológicas, Universidade Federal do Pará, Belém 66075-110, Brazil; givagosouza@gmail.com; 5 Faculty of Rehabilitation, Józef Piłsudski University of Physical Education in Warsaw, 00-968 Warsaw, Poland

**Keywords:** neuromuscular warm-up, eccentric hamstring, dynamic stretching, injury prevention, dose–response, youth sport, sprint/jump performance, training load, ramp-up, recovery

## Abstract

Background: Targeted training interventions, including neuromuscular warm-up protocols, eccentric resistance training, and dynamic stretching exercises, with limited and heterogeneous evidence on recovery strategies, have shown potential benefits for muscle performance and reductions in injury risk. Objective: To synthesize and structure contemporary evidence on exercise-based interventions spanning the pre-activity warm-up through post-activity recovery, emphasizing performance outcomes, injury-related effects, reported dose–response patterns, and implementation characteristics. Methods: This systematic review with structured qualitative synthesis was based on a systematic database search and eligibility screening process (*n* = 40 studies). Studies evaluating exercise-based interventions targeting injury prevention and/or performance in athletes were included. Data extraction included study design, population characteristics, intervention components, outcomes, and risk-of-bias assessments, which were summarized using comparative tables and descriptive analyses. Due to heterogeneity, results were synthesized qualitatively without meta-analysis. Results: Neuromuscular warm-ups (e.g., FIFA 11+) were frequently linked to reduced reported lower-extremity injury risk, alongside improvements in sprint, jump, and balance performance. Eccentric hamstring training was linked in several studies to lower reported hamstring injury rates and increased eccentric strength and fascicle length, while dynamic warm-ups may acutely improve sprint and jump performance. Evidence regarding recovery modalities was heterogeneous, supporting a cautious and individualized emphasis on sleep, nutrition, and hydration. However, heterogeneity in study designs, outcome measures, and populations limits the comparability and generalizability of the findings. Conclusions: Exercise-based interventions, including neuromuscular warm-ups, eccentric strengthening, dynamic stretching, and progressive load and recovery strategies, may support performance enhancement and injury-risk reduction in youth and adult athletes when appropriately implemented, although interpretation is limited by study heterogeneity.

## 1. Introduction

Muscle–tendon unit injuries, such as muscle strains and tendinopathies, often arise from the accumulation of fatigue, inadequate preparation for exercise, and abrupt fluctuations in training load. Exercise-based interventions implemented in training practice, particularly structured neuromuscular warm-ups, eccentric hamstring training, dynamic mobility exercises, and controlled load management, are generally associated with improved sprinting, jumping, and balance performance, as well as with meaningful reductions in lower-extremity injuries [1,2,3,4,5,6,7]. School- and club-level programs, such as FIFA 11+ and iSPRINT, demonstrate high scalability and adherence [8,9], while targeted eccentric protocols (e.g., the Nordic hamstring exercise) effectively reduce the risk of hamstring injuries [10,11]. Dynamic warm-ups help maintain or enhance explosive performance compared with prolonged static stretching, in line with the current consensus on pre-exercise preparation [12,13]. Pre-conditioning strategies and the use of wearable resistance may enhance short-term readiness [14,15], whereas structured injury-prevention programs have been shown to reduce severe knee injuries in elite football players [16,17]. Existing reviews most often address warm-up protocols, strength interventions, or recovery strategies separately, with limited integration across the entire training–competition continuum [18,19,20]. Moreover, performance enhancement and injury prevention are frequently treated as distinct objectives, despite sharing common neuromuscular and load-related determinants. However, a comprehensive synthesis integrating these strategies across the full training–recovery continuum remains lacking.

This review aims to integrate evidence across the warm-up, training, load management, and recovery phases through a structured qualitative synthesis aimed at identifying general patterns across intervention types within neuromuscular, performance, and load-related domains, without conducting direct comparative effectiveness analyses or formal evaluation of interaction effects and without performing a pooled quantitative analysis.

## 2. Materials and Methods

### 2.1. Protocol Registration and Reporting Guidelines

This systematic review with qualitative synthesis was conducted according to the Preferred Reporting Items for Systematic Reviews and Meta-Analyses (PRISMA) 2020 [4] guidelines and was prospectively registered in PROSPERO (Registration ID: CRD420251154465). The protocol followed PRISMA-P recommendations for protocol development.

### 2.2. Design and Sources

This systematic review was conducted following the Preferred Reporting Items for Systematic Reviews and Meta-Analyses (PRISMA) 2020 guidelines. The review was based on an evidence set comprising 40 human studies, including randomized controlled trials (*n* = 11), cluster randomized controlled trials (*n* = 3), meta-analyses (*n* = 12), systematic reviews (*n* = 7), controlled/quasi-experimental studies (*n* = 4), cohort studies (*n* = 2), and observational studies (*n* = 1), identified through the systematic database search and eligibility screening process described below. To comprehensively characterize the available evidence base, a structured qualitative synthesis approach was employed, integrating both primary studies and secondary evidence (systematic reviews and meta-analyses). Such an approach facilitates the translation of evidence into practice through a combined analysis of the data. Given the heterogeneity in study designs, populations, and outcome measures, a quantitative meta-analysis was not performed, and findings were synthesized narratively.

An extensive literature search was conducted across multiple databases, including PubMed, Scopus, Web of Science, EBSCO, and SPORTDiscus, from database inception through August 2025. The search strategy was developed around the guiding research question “From Warm-Up to Recovery: Optimizing Muscle Performance and Injury Prevention through Training Interventions” and employed a combination of Medical Subject Headings (MeSH) terms and free-text keywords. Search terms included combinations of: (“neuromuscular training” OR “warm-up” OR “injury prevention” OR “eccentric training” OR “strength training” OR “FIFA 11+” OR “iSPRINT”) AND (“muscle performance” OR “injury incidence” OR “athletic performance” OR “sprint” OR “jump” OR “balance”) AND (“randomized controlled trial” OR “systematic review” OR “meta-analysis”). No language or date restrictions were applied to maximize comprehensiveness. The full search strategy for each database is provided in the Supplementary Table S7.

**Figure 1:**
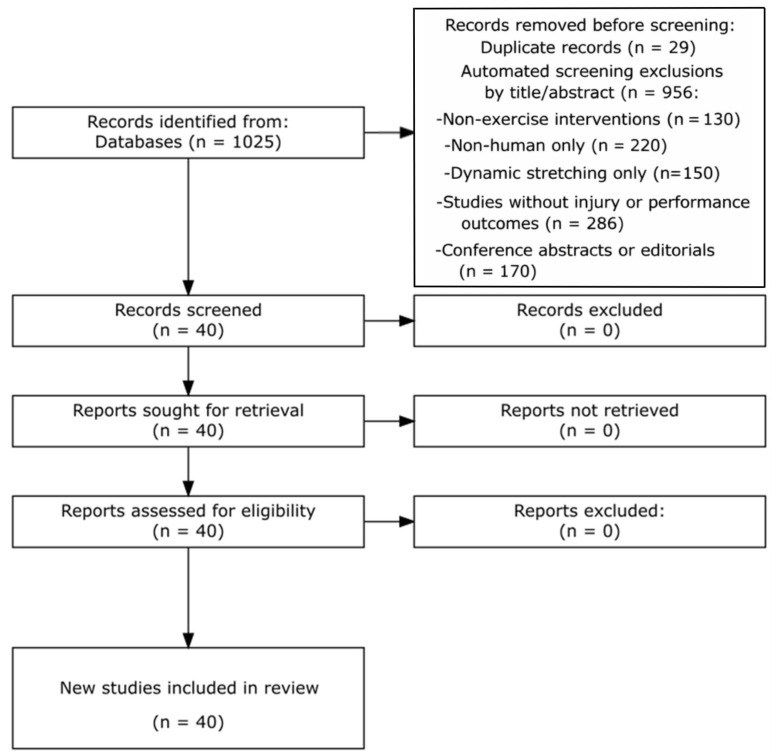
PRISMA flow diagram illustrating the study selection process.

Study Selection Process: The initial database searches yielded 1025 records. After the automated removal of 29 duplicate records using reference management software (EndNote 20, Clarivate Analytics, Philadelphia, PA, USA), with duplicate detection based on matching author names, titles, publication years, and DOIs, 996 records underwent preliminary screening based on titles and abstracts. During this phase, 956 records were excluded using predefined automated criteria: non-exercise interventions (*n* = 130), non-human or mechanistic studies only (*n* = 220), studies without injury or performance outcomes (*n* = 286), dynamic stretching-only interventions (*n* = 150), and conference abstracts or editorials (*n* = 170). Automated screening was limited to initial filtering, and all exclusions were verified manually by two independent reviewers. Studies focusing exclusively on dynamic stretching were excluded because such interventions do not represent comprehensive training strategies and do not align with the integrative scope of this review, which aimed to evaluate multicomponent or structured exercise-based interventions across the training–recovery continuum. Automated exclusions were verified by two independent reviewers to ensure consistency and accuracy of the screening process. This systematic screening process resulted in 40 studies that met our inclusion criteria and were included in the final qualitative synthesis (see Figure 1). To avoid double counting of evidence, findings from systematic reviews and meta-analyses were interpreted alongside primary studies without being combined quantitatively.

Quality Assessment and Data Extraction: Two independent reviewers extracted data using standardized forms, including study design, participant characteristics, intervention details, comparator conditions, outcome measures, and key statistical findings. Methodological quality was assessed using appropriate tools based on study design, with discrepancies resolved through consensus or third-reviewer consultation.

### 2.3. Eligibility Criteria

Inclusion criteria: (i) human participants engaged in sport or structured exercise; (ii) an exercise-based warm-up, training (NMT/FIFA 11+, eccentric hamstring, dynamic stretching, wearable resistance, preseason ramp-up), or recovery protocol (defined as post-exercise interventions including active recovery, nutritional strategies, and sleep-related approaches); (iii) at least one muscle performance or injury outcome (sprint time, CMJ, Y-Balance, injury incidence/rate); and (iv) a randomized/controlled design, cohort, or systematic review/meta-analysis.

We excluded non-empirical work, mechanistic studies without applied outcomes, and modalities without an exercise component. Study selection: titles and abstracts were screened independently by two reviewers, with disagreements resolved through consensus.

### 2.4. Outcomes and Definitions

Primary outcomes included injury incidence and time-loss metrics, expressed using effect measures such as relative risk (RR) and incidence rate ratio (IRR). Performance outcomes included linear sprint (5–30 m), jump height (CMJ/VJ/SLJ), agility/change of direction, Y-Balance Test (YBT), VO_2_max, and neuromuscular indices (e.g., eccentric strength, hamstring-to-quadriceps ratio, fascicle length). Subgroup effects (sex, age) and dosing (frequency, duration, weekly exposure, intervention length) were abstracted [2,12].

### 2.5. Data Extraction and Reliability

Two reviewers independently extracted design, participant characteristics, intervention descriptors, comparators, primary outcomes (definitions, tools, timing), and key statistics (effect sizes, confidence intervals, *p*-values, RRs/IRRs). Discrepancies were resolved by consensus or third-reviewer adjudication. Inter-rater agreement was quantified using Cohen’s kappa coefficient, indicating moderate agreement (κ = 0.44) according to Landis and Koch criteria.

### 2.6. Risk of Bias

Methodological quality and risk of bias were independently assessed by two reviewers using design-specific validated tools [21,22,23]. For randomized controlled trials (RCTs) and cluster-RCTs, the Cochrane Risk of Bias 2 (RoB 2) tool was applied. Non-randomized and observational studies were evaluated using the ROBINS-I tool. Included systematic reviews and meta-analyses were appraised using the AMSTAR 2 checklist.

Domains assessed included the randomization process, allocation concealment, deviations from intended interventions, missing outcome data, outcome measurement, and selective reporting. Each study was categorized as low risk, some concerns, or high risk of bias.

Disagreements between reviewers were resolved by consensus, and when necessary, consultation with a third reviewer. A summary of methodological quality assessments is provided in Supplementary Table S2.

Given the heterogeneity of study designs and outcome metrics, risk-of-bias ratings were incorporated qualitatively into the interpretation of findings rather than used to weight pooled estimates.

### 2.7. Synthesis and Supplementary Analyses

The synthesis was conducted according to intervention class, defined based on the primary training modality (e.g., neuromuscular warm-up, eccentric training, multicomponent programs, and load management strategies) and population (e.g., youth vs. adult athletes, sex-specific cohorts, and sport type where reported), presenting comparative tables covering the spectrum of study designs, injury-risk metrics, performance outcomes, neuromuscular adaptations, sex-specific patterns, and dose–response relationships. Given heterogeneous denominators and metrics, we computed descriptive summaries (medians and ranges of reported effect estimates and performance changes) rather than a pooled meta-analysis [1].

## 3. Results

### 3.1. Evidence Base and Study Characteristics

The final included 40 studies: meta-analyses (*n* = 12), randomized controlled trials (*n* = 11), systematic reviews (*n* = 7), controlled/quasi-experimental studies (*n* = 4), cluster RCTs (*n* = 3), cohort studies (*n* = 2), and observational studies (*n* = 1). Soccer/football predominated, with basketball, rugby, and mixed-sport cohorts also represented. Youth/adolescent samples were frequent, including female-only and male-only cohorts [2,21] (see Figure 1). Table 1 summarizes the distribution and intervention classes. Supplementary Table S4 summarizes populations, interventions, dose/exposure, and primary outcomes extracted from the extended dataset.

### 3.2. Effects on Injury Outcomes

**Figure 2:**
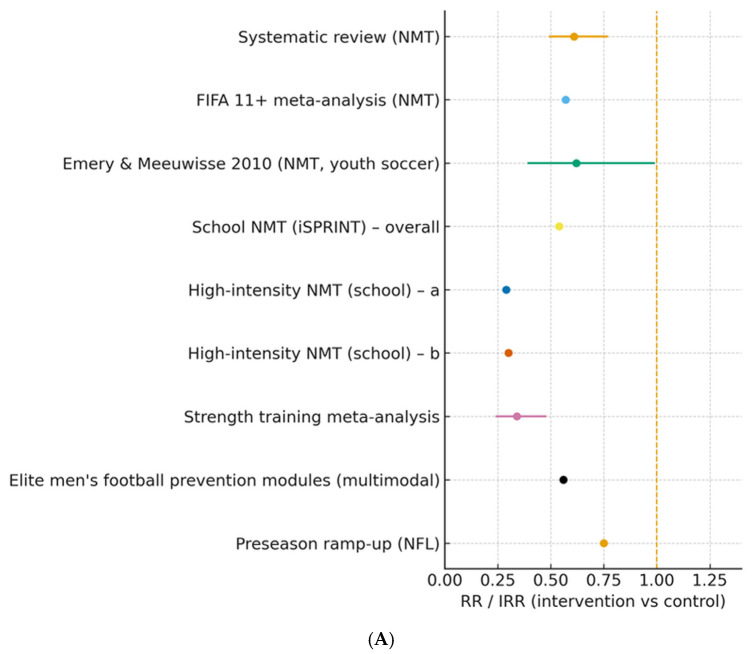

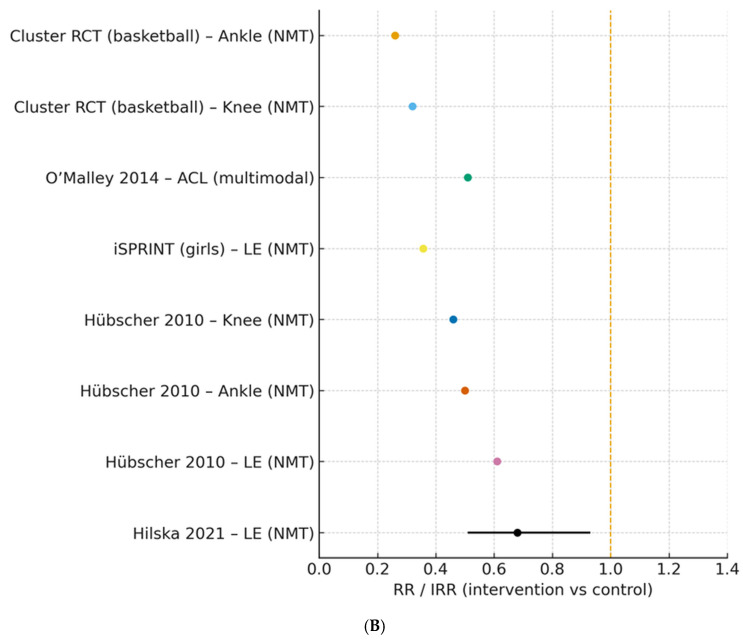
(**A**) Class-stratified forest plot of study-level injury-risk metrics (RR/IRR) across neuromuscular warm-ups (NMT/FIFA 11+), eccentric training interventions (including Nordic hamstring exercise, where applicable), multicomponent programs, and preseason ramp-up strategies; estimates expressed as intervention vs. control (values < 1.0 favor intervention), with 95% CIs shown where reported, and comparator directions harmonized. (**B**) Forest plot of joint-/compartment-specific injury-risk metrics (ankle, knee, ACL, lower extremity) from representative randomized trials and reviews; conventions as in Figure 2A (values < 1.0 favor intervention; 95% CIs where reported; NR indicates not reported). RR expresses the ratio of event risk between intervention and control groups, whereas IRR compares event incidence rates while accounting for exposure time [23,25,26].

Across NMT/FIFA 11+ programs, estimates from meta-analyses and individual trials generally favored the intervention (RR ≈ 0.57–0.73; several IRRs < 0.60), suggesting reductions in ankle and knee injury risk. Eccentric hamstring programs reduced hamstring injury, including recurrent cases, and improved eccentric strength. Refer to Figure 2A,B for class-stratified and joint-specific forest plots, and to Supplementary Table S5 for representative injury-risk estimates with harmonized comparator direction [4,21,23,24].

#### 3.2.1. Injury Prevention: Detailed Effects from Extended Dataset

##### Overall Signal and Consistency

Across studies (*n* = 26 comparisons), injury risk was generally lower in intervention versus control groups, with more pronounced effects observed for structured neuromuscular warm-ups and eccentric hamstring work. For neuromuscular training (NMT; including FIFA 11+, iSPRINT, and high-intensity NMT), non-weighted descriptive summaries of reported effect estimates yielded a median risk ratio of approximately 0.59, with a range of 0.26–0.73. These values suggest relative risk reductions of around 41%, with several trials reporting stronger effects at joint-specific endpoints (Figure 2A) [7,8,23,26]. Effects were directionally consistent in meta-analyses and large cluster RCTs, and persisted when analyses were stratified by age, sex, and sport [8,22,27].

##### By Intervention Class

- NMT/FIFA 11+. Estimates generally favored intervention across school-based and sport-specific cohorts, with similar magnitudes in meta-analyses and individual trials. Effects appeared more pronounced for joint-specific outcomes (ankle, knee) and in female cohorts, although reductions were also reported in youth male populations [9,28].
- Eccentric hamstring (NHE). Clinically meaningful reductions in hamstring injuries were reported, including recurrent cases [10,11,29]. NNT estimates indicate practical relevance in applied settings.
- Multicomponent programs. Balance- and agility-focused interventions demonstrated reduced injury rates in team-sport contexts, including elite football cohorts [16,25,30].
- Ramp-up/load management. Data-driven preseason ramp-up strategies were associated with reduced strain injuries, supporting progressive exposure models [19,31].

##### Link to Visualization

Class-stratified estimates and representative trial points are displayed in Figure 2A,B (panel A: global NMT/NHE/multicomponent summary; panel B: joint-specific endpoints). We annotate directionality where original reports used differing numerators/denominators and flag NR entries (see footnotes to Table 2).

### 3.3. Effects on Performance and Neuromuscular Adaptations

Performance gains co-occurred with preventive effects. FIFA 11+-type programs were associated with improvements in vertical jump (~+4.7 cm) and reduced 20 m sprint (~−0.38 s). Elite female basketball NMT improved CMJ (~+9.4%) and Y-Balance (~+3–4%). Eccentric-overload work in junior soccer was associated with moderate-to-large CMJ effects and positive sprint responses. Wearable-resistance and high-intensity warm-ups showed sport-specific sprint/jump effects [3,8,13,15,28,33,37]. See Supplementary Table S6 and Table 3.

Training-induced changes included increased hamstring eccentric strength, longer biceps femoris fascicle length, favorable hamstring-to-quadriceps ratios, reduced inter-limb asymmetry, improved postural control, and reduced EMG-based delay times mechanistic correlates plausibly mediating injury-risk reduction [5,28,29,34,37,40]. Table 4 summarizes reported neuromuscular adaptations.

### 3.4. Moderators of Response

Two school-based iSPRINT trials reported protective effects in girls but not boys; a female-specific analyses reported substantial ACL and ankle reductions; one youth multimodal program observed greater performance effects in boys [5,9,28,30]. Table 5 collates exemplars.

### 3.5. Intervention Characteristics

**Figure 3:**
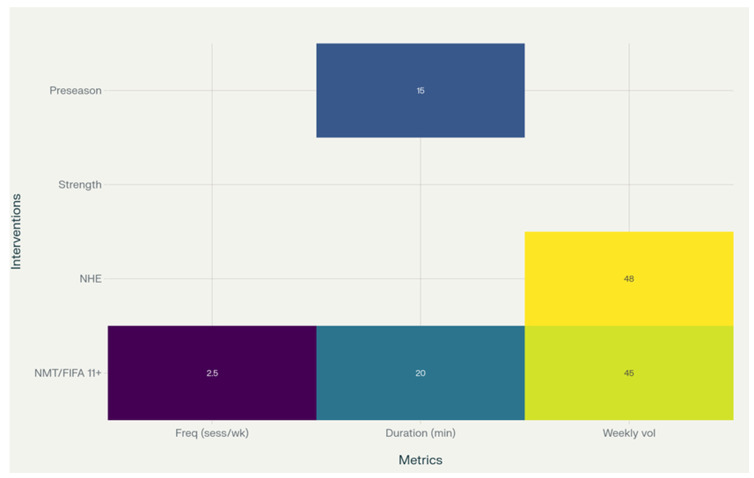
Schematic, descriptive summary of intervention characteristics (frequency, session duration, and weekly volume) derived from the included studies across neuromuscular warm-ups, Nordic hamstring exercises, strength training, and preseason ramp-up strategies. This figure does not represent a quantitative dose–response analysis or pooled estimates.

Evidence from multiple studies indicates that neuromuscular training (NMT) programs such as the FIFA 11+ are typically delivered two to three times per week for sessions lasting approximately 10–30 min (total weekly exposure ≈ 30–60 min). One randomized trial reported no significant difference in protective effect between 10- and 20-min sessions. Nordic hamstring exercise protocols generally employ a high-volume introductory phase followed by maintenance of ≈48 repetitions per week. A meta-analysis of strength-training interventions reported that a 10% increase in training volume is associated with an approximate 4% reduction in injury risk, and in professional preseason contexts, progressive ramp-up strategies were associated with reductions in lower-extremity strain injuries by around 25% [4,11,27,31,35,38]. See Figure 3 and Table 6.

#### Dose and Delivery

Preventive effects were typically delivered as brief, frequent exposures: 2–3 sessions·wk^−1^, 10–30 min·session^−1^ (30–60 min·wk^−1^) for NMT [27], with no additional benefit of 20 versus 10 min in a cluster trial [38]. For NHE, front-loaded volume followed by low-dose maintenance sustained protection [11]. Further details on populations, interventions, and dosing parameters are available in Supplementary Table S4.

Comparisons of active versus passive recovery and citrulline malate versus placebo in trained cohorts reported no consistent differences for most performance outcomes, which is consistent with an individualized approach to recovery emphasizing sleep, nutrition, hydration, and athlete preference [41]. Post-match recovery responses varied depending on the type of activity performed [18,42].

## 4. Discussion

### 4.1. Principal Findings

The strength of evidence varies across study designs, with meta-analyses and randomized controlled trials providing relatively stronger support, while observational studies offer contextual insights but with greater variability. The available evidence suggests that exercise-based warm-up protocols and targeted strengthening interventions may help reduce injury risk—particularly for lower-extremity injuries—while also supporting functional performance across diverse populations, from adolescents to elite adult athletes. Evidence from meta-analyses and randomized controlled trials suggests that neuromuscular training (NMT), exemplified by the FIFA 11+ program, has been linked to protective effects, with randomized trials reporting relative risk reductions in ankle, knee, and anterior cruciate ligament (ACL) injuries in the range of 43–50% [8,24,26,32,35]. Related findings have also been reported for specific eccentric hamstring protocols, which address the high load of hamstring strains characteristic of many field sports, primarily through mechanisms involving increased fascicle length and eccentric strength [3,10,29]. Some evidence suggests dose-related effects of strength training, with incremental volume increases associated with approximately 4% reductions in injury risk, highlighting the importance of tailored and progressive loading regimens [4]. Such findings are supported by both systematic reviews and primary studies, suggesting that modifications in training volume, intensity, and cadence may influence injury risk, emphasizing the relevance of structured progressions such as pre-season ramp-ups [27,31,38].

From a practical perspective, reported reductions in absolute injury burden may support the applied relevance of these interventions. Where count data were available, absolute rate reductions appeared notable. For example, in elite female basketball, injuries were 32 vs. 79 in NMT versus control groups over a season [37]. Hamstring-specific endpoints yielded NNT values in the low teens (any injury) and single digits (recurrent), indicating potentially relevant applied benefit [10]. These magnitudes, combined with low time requirements (10–20 min integrated into warm-ups), may offer practical value for teams and school-based settings.

Additionally, post-match active recovery compared with sport-specific training produced context-dependent fatigue responses, suggesting that day-after recovery strategies may need to be individualized [18,42].

### 4.2. Mechanistic Plausibility

The biological plausibility of these preventive strategies is consistent with mechanistic insights into neuromuscular control, tissue adaptive capacity, and biomechanical optimization [40,43,44]. Improved motor control and postural stability, facilitated by neuromuscular training, are considered plausible contributors to reduced injury susceptibility, particularly for ligamentous injuries like ACL tears and ankle sprains [5,14]. Enhancing eccentric strength and fascicle length, through protocols like eccentric calf training or Nordic hamstring exercises, may improve the musculotendinous unit’s capacity to absorb shocks and reduce strain [13,18]. A related factor is the balance of antagonist and agonist muscle forces, specifically the H:Q ratio, where normalization can decrease anterior shear forces on the knee during dynamic tasks [37]. These tissue-level adaptations may also be complemented by dynamic warm-up routines and priming activities, which can increase neuromuscular excitability and facilitate rapid activation prior to exertion [29]. Additionally, the reduction in ACL injury risk observed particularly among female athletes may be partly explained by targeted neuromuscular training that improves landing mechanics and proprioception, aligning with findings from meta-analyses [8,30]. Biomechanical studies indicate that enhanced control of joint kinematics and kinetics can reduce abnormal stress on vulnerable structures, thereby offering a plausible mechanistic foundation for the observed clinical outcomes. For detailed quantitative dosing targets relevant to these mechanisms, see Supplementary Table S1a–g.

### 4.3. Load Management and Ramp-Up

Central to injury prevention is the principle of avoiding abrupt load spikes, which can overwhelm tissue capacity and precipitate injury [1,19]. Structured preseason ramp-up protocols, typically involving progressive increases in session duration and intensity over approximately 15-minute exposures, have been associated with reductions in lower-extremity strains by up to 25% in high-performance athletes, such as professional football players [31]. This is supported by findings indicating that gradual load increments and burden modulation may reduce the risk of overuse injuries by allowing connective tissue and neuromuscular systems to adapt safely [17]. Similarly, policy-level interventions, such as implementing injury screening and early detection programs (PPE), can further optimize training loads and prevent cumulative microtrauma [16]. When integrated into broader training frameworks, these strategies appear consistent with patterns of improved readiness and lower injury risk reported across studies.

### 4.4. Warm-Up Strategies, Priming, and Wearable Aids

The available literature suggests that dynamic stretching and activation exercises can be effective components of warm-up routines designed to prepare the neuromuscular system for explosive activity [12,13]. Static stretching, particularly when prolonged (>60 s), has been associated with subsequent performance decrements and may be more appropriate for flexibility-focused sessions than for pre-activity routines [13]. The concept of post-activation potentiation (PAP), whereby brief, high-intensity plyometric or resistance exercises may enhance neuromuscular output, appears promising when carefully dosed to avoid fatigue and overload [14,18,45]. Emerging pilot studies utilizing wearable resistance devices during warm-up have shown promising results in improving sprint and jump metrics, suggesting a potential adjunct to traditional warm-up protocols [15].

### 4.5. Implementation, Adherence, and Gender-Specific Considerations

Effective deployment of prevention programs hinges not only on their scientific efficacy but also on adherence, fidelity, and contextual tailoring. Embedding 10–20-minute neuromuscular training sessions into daily routines, along with structured checklists and progressively phased protocols, such as combined eccentric and isometric exercises, has been associated with better compliance among athletes [22,26]. Notably, data from school-based interventions indicate that girls derive relatively stronger protective effects from neuromuscular training modules, while boys often exhibit larger performance gains, suggesting a potential need for gender-sensitive modifications [9]. Such tailored approaches, combined with ongoing monitoring and feedback, may help optimize long-term adherence and maximize the protective effects reported in controlled studies.

### 4.6. Limitations and Generalizability

Heterogeneous denominators (per 1000 h vs. per season), effect metrics (RR vs. IRR), and varied protocols complicate pooling. Some large-scale meta-analyses and cluster RCTs strengthen confidence [4,36], but smaller or incompletely reported studies temper certainty. Most evidence involves youth soccer, with fewer studies in other sports or older adults, limiting generalizability [15,19].

In addition, some inconsistencies across studies were observed. Considering the broader body of evidence from randomized studies and synthesized analyses [4,8,9,11,24,25], these discrepancies may be related to differences in data coding or the selection of non-comparable control groups within individual studies. Importantly, excluding the most divergent result did not substantially change the overall pattern observed in our descriptive synthesis. Studies with unreported *p*-values (NR) [31] were retained in the qualitative synthesis but interpreted with caution.

### 4.7. Future Directions

Comparative effectiveness trials of NMT content variants, maturation-sensitive eccentric dosing, pragmatic load monitoring, and digital adherence supports are warranted. Broader sport representation and standardized reporting of denominators and fidelity would enhance translation.

### 4.8. Practical Recommendations

Available evidence suggests that commonly implemented intervention strategies include structured neuromuscular warm-up protocols lasting approximately 10–20 min, incorporating balance exercises, landing control, trunk–hip stability, progressive plyometrics, and short accelerations [2]. Eccentric hamstring training is typically performed one to three times per week, often with an initial progressive loading phase, and may be integrated with sprint-specific drills [10,11].

Dynamic stretching is frequently used as part of pre-activity preparation, whereas prolonged static stretching is more often reserved for flexibility-focused sessions [12,13]. Load management strategies generally involve monitoring weekly training volume and intensity, with gradual increases during preseason periods rather than abrupt spikes [1,31]. Recovery strategies primarily emphasize sleep, nutrition, and hydration, with mixed evidence regarding the effectiveness of active versus passive modalities, depending on context and athlete characteristics [18,41,42]. These implementation patterns are broadly aligned with the approaches described in studies reporting favorable injury-related outcomes.

## 5. Conclusions

Available evidence suggests that neuromuscular warm-ups performed 2–3 times per week for 10–20 min, phased eccentric hamstring training with low-volume maintenance, dynamic rather than prolonged static stretching prior to explosive tasks, progressive preseason load management, and individualized recovery strategies may represent practical components of exercise-based programs for supporting performance and lower-extremity injury prevention in youth and adult athletes. However, these conclusions should be interpreted in light of heterogeneity in study designs, outcome measures, and athlete populations [4,8,10,11,36,41].

## Figures and Tables

**Table 1:** Structural features of the evidence base: study designs and intervention categories.

Category	*n*
Meta-analysis	12
Randomized controlled trial (individual)	11
Systematic review	7
Controlled/quasi-experimental	4
Cluster RCT	3
Cohort	2
Observational	1
Neuromuscular warm-up (NMT/FIFA 11+)	25
Eccentric hamstring/Nordic (NHE)	9
Stretching (static/dynamic/PNF)	8
General warm-up (non-NMT)	4
Load monitoring (incl. quantification)	4
Multimodal prevention program	3
Gradual “ramp-up” strategy	1
Wearable resistance	1

Abbreviations: NMT = neuromuscular training; NHE = Nordic hamstring exercise; RCT = Randomized Controlled Trial; FIFA = Fédération Internationale de Football Association.

**Table 2:** Injury prevention outcomes: extended effects.

Study	Intervention	Population	Effect Size/Metric	Significance
de Hoyo et al., 2015 [3]	Eccentric-overload	Junior elite soccer	ES = 0.94 (days absence); lower severity/incidence	Likely/possible effects
Lauersen et al., 2018 [4]	Strength training	12–40 years athletes	RR = 0.338 (0.238–0.480)	*p* < 0.0001
Grooms et al., 2013 [6]	F-MARC 11+	Male collegiate soccer	RR = 0.28 (0.09–0.85)	*p* < 0.01
Emery & Meeuwisse, 2010 [32]	NMT	Youth soccer	IRR = 0.62 (0.39–0.99)	*p* = 0.045
Rudisill et al., 2022 [29]	Eccentric training	Adult athletes	56.8–70% ↓ hamstring injury	Significant
Richmond et al., 2017 [33]	iSPRINT NMT	Junior high	IRR = 0.48 (all); 0.38 (LE); 0.09 (time loss)	Significant
Steib et al., 2017 [27]	NMT	Youth athletes	IRR = 0.58 (0.47–0.72)	Significant
Paravlic et al., 2024 [34]	NMT warm-up	Adolescent basketball	IRR (control vs. int.) = 2.6 (10.9% vs. 23.3%)	Significant
O’Malley et al., 2014 [25]	Multifaceted	Team sports	RR = 0.65 (overall); 0.51 (ACL)	*p* = 0.03
Vlachas & Paraskevopoulos, 2022 [8]	FIFA 11+	Footballers	RR = 0.57 (0.45–0.60)	*p* < 0.01
Li & Zhu, 2025 [35]	NMT	Adolescents, male	RR = 0.73 (0.67–0.79)	Significant
Stojanović et al., 2022 [36]	Multicomponent NMT	Basketball	IRR = 0.26 (ankle); 0.32 (knee)	*p* = 0.02 (ankle)
Bonato et al., 2018 [37]	Bodyweight NMT	Elite female basketball	32 vs. 79 injuries	*p* = 0.006
Emery et al., 2019 [9]	iSPRINT NMT	Junior high	IRR = 0.543 (all, girls); 0.357 (LE, girls)	Significant (girls)
Hilska et al., 2021 [26]	NMT warm-up	U11–U14 soccer	IRR = 0.68 (0.51–0.93)	*p* = 0.014
Krutsch et al., 2019 [16]	5-module prevention	Elite football	0.38 vs. 0.68/1000 h	*p* < 0.05
Bullock et al., 2025 [30]	NMT + policy	Female athletes	LE −19%; ankle −39%; ACL −61%	Significant (ACL)
Schache, 2012 [10]	Eccentric hamstring	Male soccer	NNT = 13 (any); 25 (new); 3 (recurrent)	Significant
Rahlf et al., 2020 [38]	NMT 10 vs. 20 min	Male soccer	RR = 1.03 (0.59–1.79)	Not significant
Herzog et al., 2023 [31]	Gradual ramp-up	NFL	~25% ↓ LE strains	NR
Berg et al., 2021 [28]	iSPRINT NMT	School	IRR = 0.543 (girls, all); 0.357 (girls, LE)	Significant (girls)
Lopes et al., 2019 [39]	Eccentric; NMT	NR	RR = 3.49 (eccentric); 2.73 (NMT)	*p* < 0.00001
Herman et al., 2012 [24]	NMT warm-up	Mostly female	RR = 0.67 (11+); 0.18 (PEP, ACL)	Significant
Hübscher et al., 2010 [23]	NMT; balance	Adolescents/young adults	RR = 0.61 (LE); 0.46 (knee); 0.50 (ankle)	*p* < 0.01
Emery et al., 2015 [22]	NMT	NR	IRR = 0.64 (LE)	Significant

Abbreviations: NR = Not Reported; LE = Lower Extremity; ACL = Anterior Cruciate Ligament; PEP = Prevent injury and Enhance Performance; NMT = Neuromuscular Training; IRR = Incidence Rate Ratio; RR = Relative Risk; NNT = Number Needed to Treat; NFL = National Football League.

**Table 3:** Performance outcomes in the extended dataset.

Study	Intervention	Population	Performance Outcomes	Significance
de Hoyo et al., 2015 [3]	Eccentric-overload	Junior elite soccer	20 m sprint ES = 0.37; 10 m flying ES = 0.77; CMJ ES = 0.79	Substantial improvement
Faude et al., 2017 [5]	Multimodal program	Youth athletes	g = 0.22 (leg power); g = 0.80 (sprint); g = 0.83 (skills)	Significant
Vlachas & Paraskevopoulos, 2022 [8]	FIFA 11+	Footballers	+4.67 cm VJ; −0.38 s 20 m	Significant
Bonato et al., 2018 [37]	Bodyweight NMT	Elite female basketball	+9.4% CMJ; +4.4% YBT (R); +3.0% YBT (L)	*p* < 0.0001/0.001/0.003
Bustos et al., 2020 [15]	Wearable resistance	Soccer	Sprint ES = −1.06 to −0.96; SLJ ES = 0.85/0.93	NR
Richmond et al., 2011 [33]	High-intensity NMT	School youth	+2.14 mL/kg/min VO_2_max; +4.16 cm VJ	*p* = 0.0001/0.0003
Berg et al., 2021 [28]	iSPRINT NMT	School	+1.2 s balance (95% CI 0.2–2.1)	Significant
Behm et al., 2016 [13]	Stretching	NR	Static −3.7%; Dynamic +1.3%; PNF −4.4%	NR

Abbreviations: ES = Effect Size; VJ = Vertical Jump; CMJ = Countermovement Jump; SLJ = Standing Long Jump; YBT = Y Balance Test; VO_2_max = Maximal Oxygen Uptake; CI = Confidence Interval; NMT = Neuromuscular Training; NR = Not Reported; PNF = Proprioceptive Neuromuscular Facilitation.

**Table 4:** Strength, morphology, symmetry, and control changes following targeted programs.

Study (Abridged)	Class	Adaptations
Rudisill et al., 2022 [29]	Eccentric	↑ Hamstring strength & fascicle length; ↑ H:Q; ↓ asymmetry
Paravlic et al., 2024 [34]	NMT warm-up	↓ Delay times in muscles (improved function)
Faude et al., 2017 [5]	Multimodal	↑ Balance/stability (g = 0.37); ↑ leg power (g = 0.22)
Bonato et al., 2018 [37]	NMT	↑ Y-Balance (*p* = 0.001–0.003)
Berg et al., 2021 [28]	NMT (iSPRINT)	↑ Dynamic balance

Abbreviations: NMT = Neuromuscular Training; H:Q = Hamstring-to-Quadriceps Ratio; g = Hedges’ g; Y-Balance = Y Balance Test.

**Table 5:** Differential responses by sex to neuromuscular and multimodal programs.

Study	Class	Sex-Specific Finding
Emery et al., 2019 [9]	NMT (iSPRINT)	Protective in girls (IRR = 0.543 overall; 0.357 LE), not boys
Berg et al., 2021 [28]	NMT (iSPRINT)	Protective in girls, not boys
Bullock et al., 2025 [30]	NMT + policy	ACL −61%; ankle −39%; LE −19% (female athletes)
Faude et al., 2017 [5]	Multimodal	Larger performance effects in boys (g = 0.27–1.02) than girls (g = 0.09–0.38)

Abbreviations: ACL = Anterior Cruciate Ligament; IRR = Incidence Rate Ratio; LE = Lower Extremity; NMT = Neuromuscular Training; iSPRINT = Injury Prevention through Sprint and Neuromuscular Training.

**Table 6:** Frequency, duration, and volume targets for protective adaptation.

Intervention	Frequency	Duration	Weekly Volume	Notes
NMT/FIFA 11+	2–3×/wk	10–30 min	30–60 min	10 vs. 20 min: no difference (Rahlf et al., 2020 [38])
Nordic hamstring (NHE)	Phase-dependent	—	~48 reps/wk (maint.)	High-volume intro; maintenance thereafter (Nunes et al., 2024 [11])
Strength training	Dose-dependent	—	—	+10% volume → ~4% injury-risk reduction (Lauersen et al., 2018 [4])
Preseason ramp-up	Progressive	~15 min	—	~25% fewer strains (Herzog et al., 2023 [31])

Abbreviations: NMT = Neuromuscular Training; FIFA = Fédération Internationale de Football Association; NHE = Nordic Hamstring Exercise; wk = Week; reps = Repetitions; maint. = Maintenance.

## Data Availability

The datasets analyzed in this study are included within the article and its Supplementary Materials. No new datasets were generated.

## Supplementary Materials

The following supporting information can be downloaded at https://www.mdpi.com/article/10.3390/sports14050187/s1: Supplementary Table S1a: CSV-derived dataset (reduced columns)—Neuromuscular Training (NMT/FIFA 11+); Supplementary Table S1b: CSV-derived dataset (reduced columns)—Eccentric/Nordic Hamstring; Supplementary Table S1c: CSV-derived dataset (reduced columns)—Strength Training; Supplementary Table S1d: CSV-derived dataset (reduced columns)—Multicomponent/Policy/Program; Supplementary Table S1e: CSV-derived dataset (reduced columns)—Load Monitoring/Quantification; Supplementary Table S1f: CSV-derived dataset (reduced columns)—Preseason Ramp-up; Supplementary Table S1g: CSV-derived dataset (reduced columns)—Other/Unspecified; Supplementary Table S2: Risk of bias and methodological quality assessment of included studies; Supplementary Table S3: PRISMA 2020 checklist; Supplementary Table S4. Characteristics of included studies (extended dataset); Supplementary Table S5. Relative and incidence rate ratios for injury outcomes in controlled evaluations; Supplementary Table S6. Sprinting, jumping, and balance responses to targeted preparatory work; Supplementary Table S7. Full Search Strategy.

## Abbreviations

The following abbreviations are used in this manuscript:

ACL	anterior cruciate ligament
CI	confidence Interval
CK	creatine Kinase
CMJ	countermovement jump
EMG	electromyography
ES	effect size
H:Q	hamstring-to-quadriceps ratio
IRR	incidence rate ratio
LE	lower extremity
MVC	maximal voluntary contraction
MVF	maximal Voluntary Force
NFL	national Football League
NHE	Nordic hamstring exercise
NMT	neuromuscular training
NR	not reported
PEP	Prevent Injury and Enhance Performance
PEE	pre-participation examination
PNF	proprioceptive Neuromuscular Facilitation
PRISMA	Preferred Reporting Items for Systematic Reviews and Meta-Analyses
PRISMA-P	Preferred Reporting Items for Systematic Review and Meta-Analysis Protocols
RCT	randomized controlled trial
ROM	range of motion
RR	relative risk
SLJ	standing long jump
VO_2_max	maximal oxygen uptake
VJ	vertical jump
Wk	week
Y	years
YBT	Y Balance Test
