# Supplementary material for: Exercise-Based Strategies from Warm-Up to Training: A Systematic Review of Performance Enhancement and Injury Prevention
Source: Sports (Basel). 2026 May 6;14(5):187. doi: 10.3390/sports14050187 (PMC13210987; doi:10.3390/sports14050187)
Supplement: Supplementary file 1 [file sports-14-00187-s001.zip › Supplementary Table S1a.pdf]

Supplementary Table S1a. CSV-derived dataset (reduced columns) — Neuromuscular Training (NMT / FIFA 11+).

Displayed columns: Title; Authors; Year; Study Design; Participant Characteristics; Intervention Type and Characteristics; Comparison/Control Conditions; Primary Outcome Measures; Key Findings and Statistical Results; Risk of Bias Assessment

Title	Authors	Year	Study Design	Participant Characteristics	Intervention Type and Characteristics	Comparison/Control Conditions	Primary Outcome Measures	Key Findings and Statistical Results	Risk of Bias Assessment
Neuromuscular training for sports injury prevention: a systematic review.	M. Hübscher, A. Zech, K. Pfeifer, F. Hänsel, L. Vogt, W. Banzer	2010	Systematic review, Meta-analysis	- Age range: Adolescent and young adult<br><br>- Population type: Athletes<br><br>- Not mentioned: Total sample size, gender distribution, specific inclusion/exclusion criteria	- Precise type of intervention: Proprioceptive/neuromuscular training, multi-intervention training, balance training alone<br><br>- Duration of intervention: Not mentioned<br><br>- Frequency of intervention: Not mentioned<br><br>- Specific protocols or techniques used: Not mentioned	Not mentioned (the abstract does not provide specific details about the control conditions)	- Specific outcomes measured: Incidence of lower limb injuries, acute knee injuries, ankle sprain injuries<br><br>- Measurement tools or methods: Relative risks (RR) and 95% confidence intervals (CI)<br><br>- Timing of outcome measurements: Not explicitly mentioned	- Multi-intervention training: RR = 0.61 (95% CI = 0.49-0.77, P < 0.01) for lower limb injuries; RR = 0.46 (95% CI = 0.28-0.76, P < 0.01) for acute knee injuries; RR = 0.50 (95% CI = 0.31-0.79, P < 0.01) for ankle sprain injuries.<br><br>- Balance training alone: RR = 0.64 (95% CI = 0.46-0.9, P < 0.01) for ankle sprain injuries; RR = 0.49 (95% CI = 0.13-1.8, P = 0.28) for overall injuries.<br><br>- Exercise interventions more effective in athletes with a history of sports injury than in those without.	Not mentioned (the abstract does not provide specific details on randomization method, blinding procedures, potential sources of bias, completeness of follow-up, or conflicts of interest)
The Effect of the FIFA 11+ on Injury Prevention and Performance in Football: A Systematic Review with Meta-Analysis	Theodosis Vlachas, E. Paraskevopoulos	2022	Systematic review, Meta-analysis	- Total sample size: Not explicitly mentioned<br><br>- Age range or mean age: 13-25 years; specific mean ages for some studies: 20 (Nuhu et al.), 13-17 (Soligard et al.), 23.5 (Impellizzeri et al.),	- Precise type of intervention: Warm-up program (FIFA 11+)<br><br>- Duration of intervention: 20 minutes<br><br>- Frequency of intervention: Typically three times per week; one study performed it five times per week	- Type of control: Alternative intervention (conventional warm-up program)<br><br>- Specific details: Control groups performed a conventional warm-up program, with	- Specific outcomes measured: Injury incidence rates, muscle strength, sprint speed, jump height, balance, proprioception<br><br>- Measurement tools or methods: Landing Error Scoring System	- Pooled RR: 0.57; 95% CI: 0.45-0.60; p < 0.01<br><br>- Reduction in injury numbers: Intervention group had fewer injuries compared to control group	- Randomization method: Assessed using PEDro scale, ensuring comparable groups.<br><br>- Blinding procedures: Partially addressed; limitations in

				20.63 (Nawed et al.), 17-20 (Daneshjoo et al.), 16.8 (Ayala et al.)<br><br>- Gender distribution: Predominantly male; one study with female athletes only (Soligard et al.)<br><br>- Population type: Football players (professional and amateur)<br><br>- Specific inclusion/exclusion criteria: Included footballers of both genders and professional/amateur status; excluded studies on different sports	- Specific protocols or techniques used: Running exercises (8 minutes), strength and plyometric exercises (10 minutes), balance exercises, followed by additional running exercises (2 minutes)	frequency and duration matching the intervention group's schedule.<br><br>- How control condition differs: The control condition involved a different warm-up program (conventional) compared to the intervention group (FIFA 11+).<br><br>- Additional control group: HarmoKnee warm-up program was used as an additional control condition in one study.	(LESS), Cybex NORM isokinetic dynamometer, SEBT test, vertical jump tests<br><br>- Timing of outcome measurements: Baseline and final follow-up assessment, varying from 8 weeks to several months	- Effect sizes:<br><br>- Reduction in LESS score: High-risk athletes -2.93, Low-risk athletes -1.3<br><br>- Time to stabilization: Reduced by 2.8%<br><br>- Core instability: Reduced by 8.9%<br><br>- Concentric strength of flexors: Increased by 3.2%<br><br>- Eccentric strength of flexors: Increased by 3.8%<br><br>- Vertical jump test score: Increased by 4.67 cm<br><br>- 20 m sprint run test: Decreased by 0.38 s	blinding researchers and coaches.<br><br>- Potential sources of bias: Subjective injury diagnosis, variable execution by coaches.<br><br>- Completeness of follow-up: Assessed using PEDro scale, aiming for 85% participation.<br><br>- Conflicts of interest: None declared.
Neuromuscular Training Warm-up Prevents Acute Noncontact Lower Extremity Injuries in Children's Soccer: A Cluster Randomized Controlled Trial	Matias Hilska, M. Leppänen, T. Vasankari, S. Aaltonen, P. Kannus, J. Parkkari, K. Steffen, U. Kujala, N. Konttinen, A. Räisänen, K. Pasanen	2021	Randomized controlled trial	- Total sample size: 1403<br><br>- Age range: 9-14 years<br><br>- Gender distribution: 280 female, 1123 male<br><br>- Population type: Competitive U11-U14 soccer players in Finland<br><br>- Specific inclusion/exclusion	- Precise type of intervention: Neuromuscular training (NMT) warm-up<br><br>- Duration of intervention: 20 minutes per session (mean session length: 25 minutes, range: 10-70 minutes)<br><br>- Frequency of intervention: 2 to 3 times per week<br><br>- Specific protocols or techniques used: 7 different	- Type of control: No intervention (standard warm-up)<br><br>- Specific details of control condition: Control teams continued their standard warm-up routines without any specific intervention or alternative warm-up.<br><br>- How control condition differs	- Specific outcomes measured: Acute soccer-related lower extremity (LE) injuries<br><br>- Measurement tools or methods: Weekly text messages to parents/guardians; structured telephone interviews with physical therapists<br><br>- Timing of outcome measurements:	- Primary statistical results: The intervention group had a significant reduction in acute noncontact LE injuries compared to the control group.<br><br>- Effect sizes: 32% reduction in acute noncontact LE injuries.<br><br>- Confidence	- Randomization method: Randomization was performed by a statistician not involved in the study.<br><br>- Blinding procedures: Statisticians and the first author were blinded.<br><br>- Potential sources of bias: Inadequate

				criteria: Excluded if having an ongoing injury preventing participation or stopped playing before follow-up	exercises focusing on motor skills and movement quality	from intervention group: The control group did not receive the NMT warm-up program, unlike the intervention group.	Weekly throughout the study period	intervals: IRR for noncontact LE injuries, 0.68 [95% CI, 0.51-0.93].<br><br>- Statistical significance: p-value for noncontact LE injuries, 0.014.<br><br>- Relative risk or other comparative metrics: IRR for all acute LE injuries, 0.82 [95% CI, 0.64-1.04].	adherence to the intervention, contamination from existing injury prevention practices, and a national knee injury prevention program affecting control groups.<br><br>- Completeness of follow-up: High response rate for injury reports and low withdrawal rate.<br><br>- Conflicts of interest: Financial support from various organizations, but no explicit conflicts mentioned.
066 Implementing a school prevention program to reduce injuries through neuromuscular Training (iSPRINT): a cluster-randomized controlled trial	C. Emery, C. Berg, S. Richmond, L. Palacios-Derflingher, C. Mckay, P. Doyle-Baker, M. Mckinlay, C. Toomey, A. Nettel-Aguirre, E. Verhagen, K. Belton, A. Macpherson, B. Hagel	2020	Randomized controlled trial (specifically, cluster-randomized controlled trial)	- Total sample size: 1,067<br><br>- Age range: 11–16<br><br>- Gender distribution: 53.7% female<br><br>- Population type: School students<br><br>- Specific inclusion/exclusion criteria: Not mentioned	- Precise type of intervention: Neuromuscular training (NMT) warm-up<br><br>- Duration of intervention: 12 weeks<br><br>- Frequency of intervention: At the beginning of each physical education class (exact frequency per week not specified)<br><br>- Specific protocols or techniques used: Not detailed in the abstract	- Type of control: Alternative intervention<br><br>- Specific details of control condition: Standard-of-practice warm-up<br><br>- How control condition differs from intervention group: The control condition is the standard warm-up routine used in schools, whereas the intervention group received a neuromuscular	- Specific outcomes measured: Injury incidence rate ratios (IRR) for all injuries, lower extremity injuries, and medically-treated injuries; predicted-VO2max, vertical jump, and single-leg eyes-closed dynamic balance on foam pad.<br><br>- Measurement tools or methods: Validated injury surveillance; predicted-VO2max, vertical jump, and single-leg eyes-closed dynamic balance on foam pad.	- Primary statistical results: iSPRINT was protective against all injuries (IRR=0.543, 95%CI; 0.295–0.998), lower extremity injuries (IRR=0.357, 95%CI; 0.159–0.799), and medically-treated injuries (IRR=0.289, 95%CI; 0.135–0.619) in girls.<br><br>- Effect sizes: IRR for all injuries in girls = 0.543; IRR for lower extremity injuries in girls = 0.357; IRR for medically-treated injuries in girls =	- Randomization method: Cluster-randomized controlled trial.<br><br>- Blinding procedures: Not mentioned.<br><br>- Potential sources of bias: Exercise fidelity differences between girls and boys.<br><br>- Completeness of follow-up: Not mentioned.<br><br>- Conflicts of

						training (NMT) program.	- Timing of outcome measurements: Baseline and 12-weeks.	0.289.<br><br>- Confidence intervals: 95%CI for all injuries in girls = 0.295–0.998; 95%CI for lower extremity injuries in girls = 0.159–0.799; 95%CI for medically-treated injuries in girls = 0.135–0.619.<br><br>- Statistical significance: Statistically significant reduction in injury incidence in girls.<br><br>- Relative risk or other comparative metrics: IRR for all injuries in girls = 0.543; IRR for lower extremity injuries in girls = 0.357; IRR for medically-treated injuries in girls = 0.289.<br><br>- Improvement in dynamic balance: Mean change in balance time favored the iSPRINT group (1.2 seconds, 95%CI; 0.2–2.1).	interest: Not mentioned.
051 Implementing a school prevention program to reduce injuries through neuromuscular training (iSPRINT): a	C. Berg, C. Emery, S. Richmond, L. Palacios-Derflingher, C. Mckay, P. Doyle-Baker, M.	2021	Randomized controlled trial (Cluster-randomized controlled trial)	- Total sample size: 1,067<br><br>- Age range: 11–16<br><br>- Gender distribution:	- Precise type of intervention: Neuromuscular training (NMT) warm-up<br><br>- Duration of intervention: 12 weeks	- Type of control: Alternative intervention<br><br>- Specific details of control condition:	- Specific outcomes measured: Injury incidence rates (all injuries, lower extremity injuries, medically-treated	- Primary statistical results: iSPRINT was protective against all injuries (IRR=0.543, 95%CI; 0.295–0.998), lower	- Randomization method: Cluster-randomized controlled trial.<br><br>- Blinding

cluster-randomized controlled trial	Mckinlay, C. Toomey, A. Nettel-Aguirre, E. Verhagen, K. Belton, A. Macpherson, B. Hagel			53.7% female<br><br>- Population type: School students<br><br>- Specific inclusion/exclusion criteria: Not mentioned	- Frequency of intervention: Not explicitly mentioned (implied to be at the beginning of each PE class)<br><br>- Specific protocols or techniques used: Not detailed in the abstract	Standard-of-practice warm-up<br><br>- How control condition differs from intervention group: The control condition is a standard warm-up, whereas the intervention group received a neuromuscular training (NMT) program.	injuries), dynamic balance, predicted VO₂max, vertical jump<br><br>- Measurement tools or methods: Validated injury surveillance, single-leg eyes-closed dynamic balance on foam pad<br><br>- Timing of outcome measurements: Baseline and 12-weeks	extremity injuries (IRR=0.357, 95%CI; 0.159–0.799), and medically-treated injuries (IRR=0.289, 95%CI; 0.135–0.619) in girls.<br><br>- Effect sizes: IRR values indicate a protective effect in girls.<br><br>- Confidence intervals: Provided for each IRR.<br><br>- Statistical significance: Statistically significant for all injuries, lower extremity injuries, and medically-treated injuries in girls.<br><br>- Relative risk or other comparative metrics: IRR values indicate relative risk reduction.<br><br>- Dynamic balance improvement: Mean balance time increased by 1.2 seconds (95%CI; 0.2–2.1) in the iSPRINT group.	procedures: Not mentioned.<br><br>- Potential sources of bias: Exercise fidelity differences between girls and boys.<br><br>- Completeness of follow-up: Not mentioned.<br><br>- Conflicts of interest: Not mentioned.
Neuromuscular Adaptations to Multimodal Injury Prevention Programs in Youth Sports: A Systematic Review with Meta-Analysis	O. Faude, Roland Rössler, E. Petushek, R. Roth, L. Zahner, L. Donath	2017	Systematic review, Meta-analysis	- Total sample size: 704 participants<br><br>- Age range: 10 to 19 years<br><br>- Gender distribution:	- Precise type of intervention: Multimodal injury prevention programs (IPP) including balance, strength, power, and agility exercises; specific programs: "11+", "The 11", "HarmoKnee", "Prevent Injury	- Type of control: No intervention, sham treatment<br><br>- Specific details of control condition: Control groups	- Specific outcomes measured: balance/stability, leg power, strength, sprint ability, sport-specific skills	- Overall effects: Small for balance/stability (g = 0.37; 95%CI 0.17, 0.58) and leg power (g = 0.22; 95%CI 0.07, 0.38);	- Randomization method: Assessed using PEDro scale; raters not blinded to study authors, place of publication, and

of Randomized Controlled Trials				8 studies with only males, 5 studies with only females<br><br>- Population type: Athletes involved in organized sports (71% soccer players)<br><br>- Specific inclusion criteria: Healthy participants up to 20 years old, involved in organized sports, with a focus on multimodal injury prevention programs<br><br>- Specific exclusion criteria: Participants outside organized sports, those with neurological or neuromuscular conditions, and those undergoing rehabilitation	Enhance Performance" (PEP).<br><br>- Duration of intervention: 4 weeks to 4.5 months.<br><br>- Frequency of intervention: Varies by study; see tables for specific details.<br><br>- Specific protocols or techniques used: Static and dynamic balance exercises, plyometrics, lower limb strength and power exercises; designed as warm-up programs lasting about 15-20 minutes.	followed a common training regime without any specific intervention; one study used a sham treatment.<br><br>- How control condition differs from intervention group: The control groups did not receive any specific injury prevention program (IPP) or intervention, unlike the intervention groups which received multimodal IPP.	- Measurement tools or methods: extraction of means and standard deviations from studies; data extraction from graphs; calculation from pre-test values and change scores<br><br>- Timing of outcome measurements: pre- and post-test	Large for sprint abilities (g = 0.80; 95%CI 0.50, 1.09) and sport-specific skills (g = 0.83; 95%CI 0.34, 1.32).<br><br>- Subgroup effects: Larger in high-level athletes (g = 0.34–1.18) compared to low-level athletes (g = 0.22–0.75); Larger in boys (g = 0.27–1.02) compared to girls (g = 0.09–0.38); Larger in older athletes (g = 0.32–1.16) compared to younger athletes (g = 0.18–0.51); Larger with more training sessions (g = 0.35–1.16) compared to fewer sessions (g = 0.12–0.38).<br><br>- Specific program effects: "11+" showed moderate to large effects; "The 11" showed negligible effects; "HarmoKnee" showed moderate effects; PEP showed moderate effects.	results.<br><br>- Blinding procedures: Not explicitly mentioned for individual studies; raters not blinded during quality assessment.<br><br>- Potential sources of bias: Slight overrepresentation of small studies with large effects in sprint abilities and sport-specific tests; potential publication bias due to exclusion of non-randomized studies and gray literature.<br><br>- Completeness of follow-up: Assessed using PEDro scale; no specific details provided.<br><br>- Conflicts of interest: Authors declare no commercial or financial relationships that could be construed as a potential conflict of interest.
Implementing a junior high school-based programme to reduce sports injuries through neuromuscular	C. Emery, C. van den Berg, S. Richmond, L. Palacios-Derflingher, C. Mckay, P. Doyle-	2019	Randomized controlled trial	- Total sample size: 1067 students<br><br>- Age range: 11 to 16 years	- Precise type of intervention: Neuromuscular training (NMT) warm-up<br><br>- Duration of intervention: 15 minutes	- Type of control: Alternative intervention<br><br>- Specific details of control condition:	- Specific outcomes measured: All recorded injuries, lower extremity injuries, medically-treated injuries, time	- Primary statistical results: The iSPRINT program was protective for all recorded injuries, lower	- Randomization method: Cluster randomized controlled trial with socioeconomic representation.

training (iSPRINT): a cluster randomised controlled trial (RCT)	Baker, M. Mckinlay, C. Toomey, A. Nettel-Aguirre, E. Verhagen, K. Belton, A. Macpherson, B. Hagel			- Gender distribution: 53.7% female, 46.3% male<br><br>- Population type: Junior high school students (grades 7-9)<br><br>- Specific inclusion criteria: Schools in the Calgary area with regular PE classes taught by at least one PE specialist and included grades 7-9<br><br>- Specific exclusion criteria: Schools that previously participated in a pilot study	- Frequency of intervention: Delivered at each physical education class over a 12-week period<br><br>- Specific protocols or techniques used: Aerobic, agility, strength, and balance exercises; facilitated by student leaders; based on the Health Action Process Approach (HAPA) model	Standard-of-practice warm-up including aerobic, static, and dynamic stretching exercises<br><br>- How control condition differs from intervention group: Does not include neuromuscular training (NMT) components or the Health Action Process Approach (HAPA) model	loss injuries, knee and ankle injuries.<br><br>- Measurement tools or methods: Injury surveillance system validated for youth sports and recreation context; assessment by a Certified Athletic Therapist.<br><br>- Timing of outcome measurements: Weekly assessments over a 12-week study period.	extremity injuries, and medical attention injuries in girls but not in boys.<br><br>- Effect sizes: IRRs for girls were 0.543 for all injuries, 0.357 for lower extremity injuries, and 0.289 for medical attention injuries. For boys, IRRs were 0.866 for all injuries, 1.055 for lower extremity injuries, and 0.639 for medical attention injuries.<br><br>- Confidence intervals: 95% CI for girls were 0.295 to 0.998 for all injuries, 0.159 to 0.799 for lower extremity injuries, and 0.135 to 0.619 for medical attention injuries. For boys, 95% CI were 0.425 to 1.766 for all injuries, 0.404 to 2.753 for lower extremity injuries, and 0.266 to 1.532 for medical attention injuries.<br><br>- Statistical significance: Statistically significant for girls but not for boys.<br><br>- Relative risk or other comparative	- Blinding procedures: Not explicitly mentioned.<br><br>- Potential sources of bias: Limited generalizability, under-reporting of injuries, recall bias in exposure data, imputation of missing data.<br><br>- Completeness of follow-up: Not explicitly detailed; data imputation used for missing exposure.<br><br>- Conflicts of interest: None reported.

								metrics: IRRs indicate the relative risk of injuries in the intervention group compared to the control group.	
A multicomponent neuromuscular warm-up program reduces lower-extremity injuries in trained basketball players: a cluster randomized controlled trial	Emilija Stojanović, A. Scanlan, D. Radovanović, V. Jakovljevic, O. Faude	2022	Randomized controlled trial	- Total sample size: 112 players<br><br>- Age range or mean age: Mean age: 21.6 years; Standard deviation: 2.5 years (intervention), 2.6 years (control)<br><br>- Gender distribution: Intervention group - Male: 42, Female: 15; Control group - Male: 43, Female: 12<br><br>- Population type: Regional-level basketball players<br><br>- Specific inclusion/exclusion criteria:<br><br>- Over 16 years of age<br><br>- More than 2 years of experience in organized basketball<br><br>- Participation in teams with at least two on-court sessions per week<br><br>- Not part of an injury prevention program or neuromuscular	- Precise type of intervention: Multicomponent neuromuscular warm-up program including running exercises with active stretching, plyometrics, balance, strength, and agility drills.<br><br>- Duration of intervention: Approximately 20 minutes.<br><br>- Frequency of intervention: Part of regular warm-up routine before training sessions (frequency not explicitly stated).<br><br>- Specific protocols or techniques used: Emphasis on core stability, hip control, and proper knee alignment; quality of movement emphasized.	- Type of control: Alternative intervention<br><br>- Specific details of control condition: Usual warm-up routine, varied across teams, included technical drills centered on dribbling, passing, catching, and shooting, as well as static stretching<br><br>- How control condition differs from intervention group: Control condition does not include running exercises combined with active stretching, plyometrics, balance, strength, and agility drills	- Specific outcomes measured: Ankle sprain incidence rate, knee injury incidence rate, non-contact lower-extremity injury incidence rate<br><br>- Measurement tools or methods: Incidence rate ratio (IRR) calculated using Poisson regression analyses<br><br>- Timing of outcome measurements: Throughout the entire basketball season	- Primary statistical results: Intervention group experienced significantly lower ankle sprain incidence rate (IRR = 0.26, 95% CI = 0.05, 0.98, p = 0.02) and a tendency toward lower knee injury incidence rate (IRR = 0.32, 95% CI = 0.03, 1.78, p = 0.07).<br><br>- Effect sizes: IRR = 0.26 for ankle sprains, IRR = 0.32 for knee injuries.<br><br>- Confidence intervals: 95% CI = 0.05, 0.98 for ankle sprains; 95% CI = 0.03, 1.78 for knee injuries.<br><br>- Statistical significance: p = 0.02 for ankle sprains, p = 0.07 for knee injuries.<br><br>- Relative risk: IRR = 0.26 for ankle sprains, IRR = 0.32 for knee injuries.	- Randomization method: Cluster randomized controlled design with teams randomly allocated to intervention or control groups.<br><br>- Blinding procedures: Not explicitly mentioned for participants or outcome assessors; teams in the intervention group could not be blinded to the new warm-up program.<br><br>- Potential sources of bias: Limited generalizability to other levels of basketball, grouping of males and females, infrequency of certain types of injuries, lack of standardization in control group warm-ups.<br><br>- Completeness of follow-up: Not explicitly mentioned, but the study spanned an

				control warm-up<br><br>- Injury-free at the start of the study					entire basketball season.<br><br>- Conflicts of interest: Not mentioned.
Dose-Response Relationship of Neuromuscular Training for Injury Prevention in Youth Athletes: A Meta-Analysis	S. Steib, A. Rahlf, K. Pfeifer, A. Zech	2017	Meta-analysis	- Total sample size: 54 to 4,546<br><br>- Age range or mean age: 12 to 18 years; mean age 14 to 17 years<br><br>- Gender distribution: Both male and female; some studies focused on one gender exclusively<br><br>- Population type: Youth athletes in structured sports programs at competitive levels (sub-elite and elite)<br><br>- Specific inclusion criteria: Participation in structured sports programs, age 21 or younger, neuromuscular training programs<br><br>- Specific exclusion criteria: Not explicitly mentioned; focus on analytical designs and specific outcomes related to lower extremity injuries	- Intervention Type: Neuromuscular training (NMT)<br><br>- Components: Strength, balance, flexibility, plyometric, speed, and agility exercises<br><br>- Duration of Sessions: Typically 15-20 minutes (range 5-90 minutes)<br><br>- Frequency: Two to three times per week (range 1-5 times)<br><br>- Intervention Period: 6 to 40 weeks<br><br>- Specific Protocols: FIFA "the 11", FIFA "11+", balance training only in some studies	- Type of control: Usual practice routine or sham exercises without specific focus on neuromuscular control<br><br>- Specific details of control condition: Control groups performed either usual practice routine or sham exercises without specific focus on neuromuscular control<br><br>- How control condition differs from intervention group: Control groups did not receive neuromuscular training, which was the focus of the intervention group	- Specific outcomes measured: Lower extremity injury incidence (muscular, ligamentous, or bony injuries)<br><br>- Measurement tools or methods: Injury rate ratio (IRR)<br><br>- Timing of outcome measurements: Over the duration of the training programs (varied in length)	- Overall risk reduction: 42% (IRR = 0.58, 95% CI 0.47–0.72)<br><br>- Training frequency: Two times per week (IRR = 0.50; 95% CI 0.29–0.86), Three times per week (IRR = 0.40; 95% CI 0.31–0.53)<br><br>- Session duration: 10–15 min (IRR = 0.55; 95% CI 0.42–0.72)<br><br>- Weekly training volume: 30-60 min (IRR = 0.45; 95% CI 0.25-0.81)<br><br>- Intervention period: No additional benefit for periods >6 months<br><br>- Statistical significance: All results are statistically significant as indicated by the confidence intervals not crossing 1	- Randomization method: The paper uses the PEDro scale to assess randomization and other methodological quality aspects.<br><br>- Blinding procedures: The paper does not explicitly mention blinding procedures for all studies, but some studies included in the meta-analysis had assessor blinding.<br><br>- Potential sources of bias: The paper notes potential bias due to heterogeneity in methodological quality and publication bias.<br><br>- Completeness of follow-up: The paper does not provide detailed information on follow-up completeness for all studies.<br><br>- Conflicts of interest: The

									authors declare no conflicts of interest.
Effects of Warming Up With Lower-Body Wearable Resistance on Physical Performance Measures in Soccer Players Over an 8-Week Training Cycle.	Aníbal Bustos, Gustavo Metral, J. Cronin, Aaron M. Uthoff, J. Dolcetti	2020	Quasi-experimental study	- Total sample size: 31<br><br>- Age range: 16-18 years<br><br>- Gender distribution: Not mentioned<br><br>- Population type: National-level soccer players<br><br>- Specific inclusion/exclusion criteria: Matched for speed	- Type of intervention: Wearable resistance training (WRT) added to a warm-up<br><br>- Duration of intervention: 8 weeks<br><br>- Frequency of intervention: 2-3 times per week<br><br>- Specific protocols or techniques used: WRT group wore 200- to 600-g loads on their calves during warm-up	- Type of control: Alternative intervention (unloaded warm-up)<br><br>- Specific details of control condition: The control group performed the same warm-up routine as the intervention group but without wearing any wearable resistance.<br><br>- How control condition differs from intervention group: The control group did not wear any additional resistance during their warm-up exercises, unlike the intervention group which wore 200- to 600-g loads on their calves.	- Specific outcomes measured: 10- and 20-m sprint times, repeated sprint ability, vertical countermovement jump (CMJ), horizontal countermovement jump (standing long jump [SLJ])<br><br>- Measurement tools or methods: Not explicitly mentioned<br><br>- Timing of outcome measurements: Pre-training, mid-training, post-training	- Primary statistical results: Wearable resistance training improved 10- and 20-m sprint times more than unloaded training.<br><br>- Effect sizes: ES for 10- and 20-m sprint times = -1.06 to -0.96; ES for CMJ = ≥ 0.45 (decrease) and ≥ 0.27 (increase); ES for SLJ = 0.85 and 0.93.<br><br>- Confidence intervals: Not mentioned.<br><br>- Statistical significance (p-values): Not mentioned.<br><br>- Relative risk or other comparative metrics: Not mentioned.	- Randomization method: Participants were matched for speed and allocated to groups, but the exact method of randomization is not specified.<br><br>- Blinding procedures: Not mentioned.<br><br>- Potential sources of bias: Not explicitly mentioned.<br><br>- Completeness of follow-up: Data collected at multiple time points, but completeness not explicitly stated.<br><br>- Conflicts of interest: Not mentioned.
PA 05-5-1993 A school-based program to reduce injuries through neuromuscular training: isprint a cluster-randomized controlled trial	C. Emery, C. Berg, S. Richmond, L. Palacios-Derflingher, A. Nettel-Aguirre, M. Mckinlay, P. Doyle-Baker, B. Hagel	2018	Randomized controlled trial	- Total sample size: 1067<br><br>- Age range: 11–15<br><br>- Gender distribution: 53.7% female, 46.3% male<br><br>- Population type: Junior high school students<br><br>- Specific	- Precise type of intervention: Neuromuscular training (NMT) warm-up program (iSPRINT)<br><br>- Duration of intervention: 15 minutes per session<br><br>- Frequency of intervention: Implemented in PE classes over 12 weeks<br><br>- Specific protocols or techniques used: Aerobic,	- Type of control: Alternative intervention<br><br>- Specific details of control condition: Standard-of-practice warm-up<br><br>- How control condition differs from intervention group: Control schools implemented	- Specific outcomes measured: Incidence rates of all injuries, medical attention injuries, lower extremity injuries<br><br>- Measurement tools or methods: Assessment by an athletic therapist blinded to study group allocation	- Primary statistical results:<br><br>- All injuries: IRR=0.73, 95% CI 0.46 to 1.16<br><br>- Medical attention injuries: IRR=0.59, 95% CI 0.42 to 0.82<br><br>- Lower extremity injuries: IRR=0.81,	- Randomization method: Cluster-randomized controlled trial<br><br>- Blinding procedures: Athletic therapist blinded to study group allocation<br><br>- Potential sources of bias: Adjusted for sex and

				inclusion/exclusion criteria: Not mentioned	agility, strength, and balance exercises	a standard-of-practice warm-up, whereas intervention schools implemented the iSPRINT NMT warm-up program with additional practical NMT components and video.	- Timing of outcome measurements: Weekly over a 12-week period	95% CI 0.56 to 1.19<br><br>- Effect sizes:<br><br>- Reduction in all injuries: IRR=0.73<br><br>- Reduction in medical attention injuries: IRR=0.59<br><br>- Confidence intervals:<br><br>- All injuries: 0.46 to 1.16<br><br>- Medical attention injuries: 0.42 to 0.82<br><br>- Lower extremity injuries: 0.56 to 1.19<br><br>- Statistical significance:<br><br>- Medical attention injuries: Statistically significant (IRR=0.59, 95% CI 0.42 to 0.82)<br><br>- Relative risk or other comparative metrics:<br><br>- Females vs. males: IRR=1.59, 95% CI 1.12 to 2.22	clustering by class; further analysis to consider program adherence<br><br>- Completeness of follow-up: Weekly visits over 12 weeks<br><br>- Conflicts of interest: Not mentioned
Rationale and implementation of anterior cruciate ligament injury prevention warm-up	Daniel P Bien	2011	Systematic review	- Population type: Female athletes<br><br>- Gender distribution: Female	- Precise type of intervention: Warm-up program including hip and hamstring training, core stabilization, plyometrics, balance, agility,	Not mentioned (the abstract does not provide information on control or	Not mentioned (the abstract does not specify primary outcome measures, measurement tools,	Not mentioned (the abstract does not provide specific statistical results or findings	Not mentioned (the abstract does not provide

programs in female athletes.				- No information on total sample size, age range, or specific inclusion/exclusion criteria	neuromuscular training with video and verbal feedback, and stretching.<br><br>- Duration of intervention: Not mentioned.<br><br>- Frequency of intervention: Not mentioned.<br><br>- Specific protocols or techniques used: Not mentioned.	comparison conditions)	or timing of outcome measurements)	related to muscle performance or injury prevention)	information on risk of bias assessment)
Prevention strategies for lower extremity injury: a systematic review and meta-analyses for the Female, Woman and Girl Athlete Injury Prevention (FAIR) Consensus.	G. Bullock, A. Räisänen, Chelsea Martin, Maitland Martin, Jean-Michel Galarneau, Jackie L. Whittaker, J. Losciale, Mario Bizzini, Mathew N Bourne, H. Dijkstra, Marc-Olivier Dubé, Alix Hayden, M. Girdwood, M. Hägglund, S. McLeod, N. Mkumbuzi, A. Mosler, M. Murphy, G. Myklebust, M. Møller, J. Ocarino, O. Owoeye, Debbie Palmer, K. Pasanen, Ebonie K. Rio, Kristian Thorborg, M. van Middelkoop, E. Verhagen, Stuart J. Warden, Matthew Whalan, Kay M. Crossley, Carolyn A. Emery	2025	Systematic review, Meta-analysis	- Total sample size: 154,561<br><br>- Age range or mean age: Not mentioned<br><br>- Gender distribution: Females/women/girls: 84,915 (55%), Males: 69,646 (45%)<br><br>- Population type: Female/woman/girl athletes<br><br>- Specific inclusion criteria: ≥1 female/woman/girl in each study group	- Precise type of intervention: Neuromuscular Training (NMT) programs<br><br>- Duration of intervention: Minimum of 10 minutes<br><br>- Frequency of intervention: Two times per week<br><br>- Specific protocols or techniques used: Lower extremity balance, strength, agility, and change of direction exercises	Not mentioned (the abstract does not specify the type of control or comparison conditions used in the studies)	- Specific outcomes measured: Lower extremity injury incidence, ankle sprains, ACL injuries<br><br>- Measurement tools or methods: Not explicitly mentioned<br><br>- Timing of outcome measurements: Not specified	- Primary statistical results: NMT programs reduced LE injuries by 19%, ankle sprains by 39%, and ACL injuries by 61%.<br><br>- Effect sizes: 19% reduction in LE injuries, 39% reduction in ankle sprains, 61% reduction in ACL injuries.<br><br>- Confidence intervals: 0.61% to 1.08% for LE injuries, 0.36% to 1.03% for ankle sprains, 0.25% to 0.60% for ACL injuries.<br><br>- Statistical significance: Significant reduction in ACL injuries.<br><br>- Relative risk or other comparative metrics: Percentage	Not mentioned (the abstract does not provide explicit information on randomization method, blinding procedures, potential sources of bias, completeness of follow-up, or conflicts of interest)

								reductions in injury rates.	
The effectiveness of neuromuscular training warm-up program for injury prevention in adolescent male basketball players	Armin H. Paravlic, Peter Bakalár, Katarina Puš, Saša Pišot, Miloš Kalc, Kaja Teraž, Luka Šlosar, Manca Peskar, U. Marušič, B. Šimunič	2024	Randomized controlled trial	- Total sample size: 275<br><br>- Age range or mean age: Mean age 15 years with a standard deviation of 1.7 years<br><br>- Gender distribution: Male<br><br>- Population type: Adolescent male basketball players<br><br>- Specific inclusion/exclusion criteria: Not mentioned	- Precise type of intervention: Neuromuscular training (NMT) warm-up program<br><br>- Duration of intervention: Three months<br><br>- Frequency of intervention: Not explicitly mentioned<br><br>- Specific protocols or techniques used: Not detailed in the abstract	- Type of control: No intervention<br><br>- Specific details of control condition: The control group followed their usual practice without any additional intervention.<br><br>- How control condition differs from intervention group: The control group did not receive the neuromuscular training (NMT) warm-up program, which was the intervention tested in the study.	- Specific outcomes measured: Injury incidence, neuromuscular function<br><br>- Measurement tools or methods: Measurements of body anthropometry, muscle contractile properties, balance<br><br>- Timing of outcome measurements: Before and after the intervention	- Injury prevalence proportion: IG: 10.9%, CG: 23.3%<br><br>- Incidence rate ratio: 2.6 (IG vs. CG)<br><br>- Improved neuromuscular function: Reduced delay times in specific muscles (no specific statistical results provided)	- Randomization method: Randomized into intervention and control groups.<br><br>- Blinding procedures: Not mentioned.<br><br>- Potential sources of bias: Not mentioned.<br><br>- Completeness of follow-up: Not mentioned.<br><br>- Conflicts of interest: Not mentioned.
Preventing lower extremity sport injury through a high intensity neuromuscular training program in a junior high school setting	S. Richmond, C. Emery, P. Doyle-Baker, A. Nettel-Aguirre	2011	Randomized controlled trial	- Total sample size: 725 (year one), 314 (year two)<br><br>- Age range: 11 to 15 years<br><br>- Gender distribution: Not mentioned<br><br>- Population type: School youth in physical education classes<br><br>- Specific inclusion/exclusion criteria: Not mentioned	- Precise type of intervention: High intensity, neuromuscular training warm-up<br><br>- Duration of intervention: 15 minutes<br><br>- Frequency of intervention: Three times per week<br><br>- Specific protocols or techniques used: Neuromuscular training warm-up (no specific exercises detailed)	- Type of control: Standard care<br><br>- Specific details of control condition: The control group warm-up was the same duration as the intervention group but consisted of standard of care components.<br><br>- How control condition differs from intervention group: The control group did not receive the high-intensity neuromuscular	- Specific outcomes measured: Sport injury (any sport injury requiring medical attention, cessation of activity, or time loss from sport)<br><br>- Measurement tools or methods: Incidence rate ratios (IRR)<br><br>- Timing of outcome measurements: Over the 12-week intervention period	- Primary statistical results: IRR for all sport injuries = 0.29 (95% CI 0.18 to 0.46), IRR for lower extremity injuries = 0.30 (95% CI 0.17 to 0.53), IRR for time loss injuries = 0.43 (95% CI 0.20 to 0.94)<br><br>- Effect sizes: Aerobic fitness improvement = 2.14 ml/kg/min (95% CI 1.17 to 2.59), Vertical jump improvement = 4.16 cm (95% CI 3.65 to 4.66)	- Randomization method: Randomized by school; method not specified.<br><br>- Blinding procedures: Not mentioned.<br><br>- Potential sources of bias: Clustering bias addressed by adjusting for class; no mention of other biases.<br><br>- Completeness of follow-up: Not explicitly mentioned.

						training program; instead, they followed standard care practices.		- Confidence intervals: As mentioned above<br><br>- Statistical significance: p=0.0001 for aerobic fitness, p=0.0003 for vertical jump<br><br>- Relative risk or other comparative metrics: IRR values indicate a protective effect of the intervention	- Conflicts of interest: Not mentioned.
Effects of Different Training Interventions on the Recovery of Physical and Neuromuscular Performance After a Soccer Match.	A. Trecroci, S. Porcelli, E. Perri, Magda Pedrali, L. Rasica, G. Alberti, S. Longo, F. Iaia	2020	Randomized controlled trial (crossover design)	- Total sample size: 9<br><br>- Age range or mean age: Mean age 17.6 years, range 17-18 years<br><br>- Gender distribution: Male<br><br>- Population type: Subelite soccer players from a semiprofessional soccer club<br><br>- Specific inclusion/exclusion criteria:<br><br>- Inclusion: Part of a U19 National league team, adequate training volume<br><br>- Exclusion: Lower-limb injuries, inadequate training volume, history of	- Soccer-Specific Training (SST):<br><br>- Duration: 60 minutes<br><br>- Frequency: Once after a match<br><br>- Protocols:<br><br>- Warm-up: 10 minutes of FIFA 111-related running drills and 5 minutes of dynamic stretching<br><br>- Small-sided games: 20 minutes (4 vs. 4 with 3 minutes of recovery)<br><br>- Tactical drills: 15 minutes for attacking and defending<br><br>- Set plays: 10 minutes for offensive and defensive<br><br>- Active Recovery (AR):<br><br>- Duration: 30 minutes	- Type of control: Alternative intervention<br><br>- Specific details of control condition: Soccer-specific training (SST) session lasting approximately 60 minutes, including warm-up, small-sided games, tactical drills, and set plays.<br><br>- How control condition differs from intervention group: SST is more intense and focused on soccer-specific skills compared to the active recovery regime (AR), which is less intense and focused on recovery.	Primary outcome measures include 30-m sprint time, repeated sprint ability (RSA), and maximum isometric voluntary force (MVF) of knee extensors and flexors. These were measured using timing gates for sprint performance and a custom-built ergometer for MVF. Assessments were conducted immediately after the match (0 hour) and 72 hours post-match (+72 hours).	- Primary statistical results: AR significantly better restored knee flexors' muscle force compared to SST ($p < 0.05$).<br><br>- Effect sizes: ES = 20.60 for knee flexors' MVF recovery in AR compared to SST.<br><br>- Confidence intervals: Not explicitly mentioned in the text.<br><br>- Statistical significance: $p < 0.05$ for knee flexors' MVF recovery in AR compared to SST; $p > 0.05$ for sprint and RSA recovery.	- Randomization method: Participants were randomly assigned to interventions.<br><br>- Blinding procedures: Not mentioned.<br><br>- Potential sources of bias: Use of subelite players, friendly matches instead of competitive ones, small sample size.<br><br>- Completeness of follow-up: Not explicitly mentioned, but the crossover design suggests complete follow-up.<br><br>- Conflicts of

				febrile illness, prescription of medications within the last 6 months	- Frequency: Once after a match<br><br>- Protocols:<br><br>- Circle drills: 15 minutes<br><br>- Dynamic stretching: 5 minutes<br><br>- Straight-line jogging: 10 minutes (8 runs of 20 seconds each with 40 seconds of walking recovery)			- Relative risk or other comparative metrics: Not explicitly mentioned in the text.	interest: Not mentioned.
Acute effects of muscle stretching on physical performance, range of motion, and injury incidence in healthy active individuals: a systematic review.	David G. Behm, A. Blazevich, A. Kay, M. McHugh	2016	Systematic review	Not mentioned (the abstract does not provide specific details about participant characteristics such as total sample size, age range or mean age, gender distribution, or specific inclusion/exclusion criteria)	- Precise type of intervention: Static Stretching (SS), Dynamic Stretching (DS), Proprioceptive Neuromuscular Facilitation (PNF) stretching<br><br>- Duration of intervention: SS ≥60 seconds per muscle group; <60 seconds per muscle group<br><br>- Frequency of intervention: Not mentioned<br><br>- Specific protocols or techniques used: Not detailed beyond general types of stretching	- Type of control: Alternative intervention<br><br>- Specific details of control condition: Comparison between static stretching (SS), dynamic stretching (DS), and proprioceptive neuromuscular facilitation (PNF) stretching.<br><br>- How control condition differs from intervention group: Each type of stretching serves as a control or comparison condition for the others.	- Specific outcomes measured: Performance changes, range of motion (ROM) improvements, injury prevention (all-cause and overuse injuries)<br><br>- Measurement tools or methods: Not explicitly mentioned<br><br>- Timing of outcome measurements: Immediately after stretching, on average 3-5 minutes after stretching	- Primary statistical results: SS reduces performance by -3.7%, DS improves performance by +1.3%, PNF reduces performance by -4.4%.<br><br>- Dose-response relationship for SS: ≥60 s (-4.6%) vs. <60 s (-1.1%).<br><br>- SS shows a moderate performance benefit (2.2%) at longer muscle lengths.<br><br>- No data on confidence intervals, p-values, or relative risk for injury prevention.	Not mentioned (the abstract does not provide explicit information on randomization, blinding, potential sources of bias, completeness of follow-up, or conflicts of interest)
Soccer-specific warm-up and lower extremity injury	Dustin R. Grooms, Thomas G. Palmer, J. Oñate, G. Myer, T. Grindstaff	2013	Cohort study	- Total sample size: 41<br><br>- Age range: 18-25 years	- Precise type of intervention: Comprehensive warm-up program (F-MARC 11+) targeting muscular strength, body kinesthetic awareness,	- Type of control: Alternative intervention (standard dynamic warm-up)	- Specific outcomes measured: Lower extremity injury risk and time lost to lower extremity	- Injury rate per 1000 exposures: 8.1 (referent season) vs. 2.2 (intervention	- Randomization method: Not applicable (cohort study design without

rates in collegiate male soccer players.				- Gender distribution: Male<br><br>- Population type: NCAA Division III male collegiate soccer players<br><br>- Specific inclusion/exclusion criteria: Not explicitly mentioned	and neuromuscular control.<br><br>- Duration of intervention: Approximately 20 minutes.<br><br>- Frequency of intervention: 5 to 6 times per week.<br><br>- Specific protocols or techniques used:<br><br>- First component: 6 running exercises with dynamic stretching and controlled perturbations.<br><br>- Second component: 6 exercises targeting strength, balance, and jump-landing control with progression.<br><br>- Third component: 3 higher-speed running drills with cutting maneuvers.<br><br>- Supervised by an athletic trainer with progressive difficulty levels.	- Specific details of control condition: Standard dynamic warm-up following National Strength and Conditioning Association guidelines, performed before every practice and game, lasting approximately 12 weeks.<br><br>- How control condition differs from intervention group: The control group did not receive the F-MARC 11+ program, which was specifically designed to target muscular strength, body kinesthetic awareness, and neuromuscular control.	injury<br><br>- Measurement tools or methods: Tracking injuries and days lost daily, injury grading (grade 1, 2, or 3), confirmation by team physician if necessary<br><br>- Timing of outcome measurements: During the entire season, with data collected daily for each exposure	season)<br><br>- Relative risk reduction: 72% (RR = 0.28, 95% CI = 0.09, 0.85)<br><br>- Effect size for days lost: 0.733 (medium to large effect)<br><br>- Statistical significance: P < .01<br><br>- Relative risk for thigh muscle strain: 95% reduction (RR = 0.05, 95% CI = 0.03, 0.09)	randomization)<br><br>- Blinding procedures: Not mentioned<br><br>- Potential sources of bias: Lack of randomization, single institution study, no accounting for previous injuries or predisposing factors<br><br>- Completeness of follow-up: Not explicitly mentioned<br><br>- Conflicts of interest: Not mentioned
THE EFFICACY OF A NEUROMUSCULAR TRAINING INJURY PREVENTION WARM-UP PROGRAM IN JUNIOR HIGH SCHOOL STUDENTS	S. Richmond, C. Berg, O. Owoeye, Qian Shi, L. Palacios-Derflingher, B. Hagel, A. Macpherson, C. Emery	2017	Cluster randomized controlled trial	- Total sample size: 429<br><br>- Age range: 11–16 years<br><br>- Gender distribution: Not mentioned<br><br>- Population type: Junior high school students<br><br>- Specific inclusion/exclusion criteria: Not mentioned	- Precise type of intervention: Neuromuscular training (NMT) warm-up program named iSPRINT<br><br>- Duration of intervention: Not mentioned<br><br>- Frequency of intervention: Not mentioned<br><br>- Specific protocols or techniques used: Not mentioned	- Type of control: Not specified (implied to be a standard or baseline condition)<br><br>- Specific details of control condition: Not mentioned<br><br>- How control condition differs from intervention group: The control group did not receive the iSPRINT NMT program	- Specific outcomes measured: All sport injuries, lower extremity injuries, time loss injuries, knee injuries<br><br>- Measurement tools or methods: Incidence rate ratios (IRRs) using Poisson regression analyses; injuries assessed by physiotherapists or athletic therapists<br><br>- Timing of outcome	- Primary statistical results: Injury rates were significantly lower in the intervention group compared to the control group.<br><br>- Effect sizes: IRR for all sport injuries = 0.48, IRR for lower extremity injuries = 0.38, IRR for crude time loss injuries = 0.09, IRR for knee injuries = 0.18.	- Randomization method: Cluster randomized controlled trial; schools were randomized into intervention or control groups.<br><br>- Blinding procedures: Not mentioned.<br><br>- Potential sources of bias: Lack of blinding procedures could be a source of bias.

							measurements: Not specified	- Confidence intervals: 95% CI for all sport injuries = 0.26–0.91, 95% CI for lower extremity injuries = 0.20–0.74, 95% CI for crude time loss injuries = 0.02–0.45, 95% CI for knee injuries = 0.05–0.66.<br><br>- Statistical significance: All mentioned IRRs are statistically significant.<br><br>- Relative risk or other comparative metrics: The intervention group had significantly lower injury rates compared to the control group.	- Completeness of follow-up: Not mentioned.<br><br>- Conflicts of interest: Not mentioned.
The preventive effects of neuromuscular training on lower extremity sports injuries in adolescent and young athletes: a systematic review and meta-analysis.	Yuda Li, Weidong Zhu	2025	Systematic review, Meta-analysis	- Population type: Adolescents and young athletes<br><br>- Age range: 12-18 years<br><br>- Gender distribution: Males<br><br>- Total sample size: Not mentioned<br><br>- Specific inclusion/exclusion criteria: Not mentioned	- Precise type of intervention: Neuromuscular training (NMT)<br><br>- Duration of intervention: 20-30 minutes per session<br><br>- Frequency of intervention: 1-2 sessions per week<br><br>- Intervention period: ≥ 6 months<br><br>- Specific protocols or techniques: Not mentioned	Not mentioned (the abstract does not specify the type of control or comparison conditions used in the studies)	- Specific outcomes measured: Incidence of mixed lower extremity injuries, knee injuries, and ankle injuries<br><br>- Measurement tools or methods: Rate ratios (RRs)<br><br>- Timing of outcome measurements: Over the course of the intervention period, most effective when exceeding 6 months	- Primary statistical result: NMT reduces overall risk of lower extremity injuries by 27%.<br><br>- Effect size: Rate ratio (RR) of 0.73.<br><br>- Confidence intervals: Overall risk reduction (0.67-0.79), mixed lower extremity injuries (0.63-0.83), knee injuries (0.62-0.84), ankle injuries (0.63-0.84).<br><br>- Statistical	Not mentioned (the abstract does not provide explicit information on randomization method, blinding procedures, potential sources of bias, completeness of follow-up, or conflicts of interest)

								significance: Implied by confidence intervals, but no specific p-values mentioned.<br><br>- Relative risk or comparative metric: Rate ratio (RR).<br><br>- Key comparative outcome: NMT most effective for knee injuries, males, and adolescents aged 12-18 years with specific training dosages.	
The Dose-Response Relationship of Neuromuscular Training to Prevent Lower Extremity Injuries in Young Soccer Players. A Cluster Randomised Controlled Trial	A. Rahlf, A. Zech	2019	Randomized controlled trial	- Total sample size: 342<br><br>- Age range or mean age: Mean age 15.4 years ± 1.7 years<br><br>- Gender distribution: All male<br><br>- Population type: Male soccer players<br><br>- Specific inclusion/exclusion criteria: Not mentioned	- Type of intervention: Neuromuscular training<br><br>- Duration of intervention: 10 minutes (INT10) and 20 minutes (INT20)<br><br>- Frequency of intervention: Twice a week<br><br>- Specific protocols or techniques used: FIFA 11+ soccer-specific warm-up program	- Type of control: Alternative intervention<br><br>- Specific details of control condition: Both groups completed the same soccer-specific warm-up program (FIFA 11+) twice a week, but for different durations: INT10 for 10 minutes and INT20 for 20 minutes.<br><br>- How control condition differs from intervention group: The difference is in the duration of the intervention, with INT10 being 10	- Specific outcomes measured: Incidence of lower extremity (LE) injuries<br><br>- Measurement tools or methods: Player exposure hours collected monthly<br><br>- Timing of outcome measurements: Monthly over six months during one soccer season	- Primary statistical results: No significant group difference in injury incidence.<br><br>- Effect sizes: Relative risk (RR) = 1.03.<br><br>- Confidence intervals: 95% CI = 0.59, 1.79.<br><br>- Statistical significance: No significant difference between groups.<br><br>- Relative risk or other comparative metrics: RR = 1.03, indicating no significant difference in injury	- Randomization method: Cluster-randomized controlled trial<br><br>- Blinding procedures: Not mentioned<br><br>- Potential sources of bias: Not mentioned<br><br>- Completeness of follow-up: Good follow-up period with monthly data collection over six months<br><br>- Conflicts of interest: Not mentioned

						minutes and INT20 being 20 minutes.		risk between 10 and 20 minutes of neuromuscular training.	
A Customized Warm-up Design And Controlled Feasibility Trial In Adult Amateur Rugby Union	Patrick Dolan, T. Comyns, Liam G Glynn, H. Purtill, Ian C. Kenny	2023	Cohort study, Controlled trial	- Total sample size: 838 players<br><br>- Age range or mean age: Not mentioned<br><br>- Gender distribution: Intervention group - 3 women's clubs, 2 men's clubs; Control group - 1 women's club, 15 men's clubs<br><br>- Population type: Adult amateur Rugby Union clubs in Ireland<br><br>- Specific inclusion/exclusion criteria: Not mentioned	- Precise type of intervention: Warm-up intervention<br><br>- Duration of intervention: Not mentioned<br><br>- Frequency of intervention: Performed before trainings and matches<br><br>- Specific protocols or techniques used: Not mentioned	- Type of control: No intervention or standard care<br><br>- Specific details of control condition: 16 clubs (1 women's, 15 men's) did not receive the customized warm-up program<br><br>- How control condition differs from intervention group: The control group did not receive the warm-up intervention designed to reduce injuries	- Specific outcomes measured: Program acceptability, appropriateness, and feasibility<br><br>- Measurement tools or methods: Likert-type scale ratings<br><br>- Timing of outcome measurements: During or after the season-long controlled trial	- Primary statistical results: 100% completely agree or agree for acceptability; 95% completely agree or agree for appropriateness; 95% completely agree or agree for feasibility.<br><br>- Effect size: Fewer hamstring strains in the intervention group (1.1/1000 player-hours) compared to the control group (4.6/1000 player-hours).<br><br>- Confidence intervals: 95% CI 0.3-4.2 for intervention; 95% CI 3.3-6.6 for control.<br><br>- Statistical significance (p-values): Not mentioned.<br><br>- Relative risk or other comparative metrics: Not mentioned.	- Randomization method: Not mentioned<br><br>- Blinding procedures: Not mentioned<br><br>- Potential sources of bias: Not mentioned<br><br>- Completeness of follow-up: Not explicitly mentioned<br><br>- Conflicts of interest: Not mentioned
Neuromuscular training reduces lower limb injuries in elite female	M. Bonato, R. Benis, A. L. Torre	2018	Randomized controlled trial	- Total sample size: 160 players<br><br>- Age range or mean	- Precise type of intervention: Bodyweight neuromuscular exercises	- Type of control: Alternative intervention	- Specific outcomes measured: Incidence of lower limb injuries, lower limb	- Primary statistical results:<br><br>- Chi-square test:	- Randomization method: Cluster randomized controlled trial

basketball players. A cluster randomized controlled trial				age: Not mentioned<br><br>- Gender distribution: Female<br><br>- Population type: Elite female basketball players<br><br>- Specific inclusion/exclusion criteria: Not mentioned	- Duration of intervention: Not mentioned<br><br>- Frequency of intervention: Not mentioned<br><br>- Specific protocols or techniques used: Not mentioned	- Specific details of control condition: Standard tactical-technical exercises before training<br><br>- How control condition differs from intervention group: The control group performed standard exercises, whereas the intervention group did bodyweight neuromuscular exercises.	strength, postural control<br><br>- Measurement tools or methods: Countermovement Jump (CMJ), composite Y-Excursion Balance test (YBT), epidemiologic incidence proportion, incidence rate<br><br>- Timing of outcome measurements: During the 2015-2016 regular season	EG vs CG injuries (32 vs 79, P = .006)<br><br>- Injuries during training: EG vs CG (21 vs 52, P < .0001)<br><br>- Injuries during matches: EG vs CG (11 vs 27, P = .006)<br><br>- Epidemiologic incidence: EG vs CG (0.37 vs 1.07, P = .023)<br><br>- Incidence rate: EG vs CG (1.66 vs 4.69, P = .012)<br><br>- Effect sizes:<br><br>- CMJ improvement: +9.4% (P < .0001; d = 1.2)<br><br>- YBT improvement: right +4.4% (P = .001, d = 1.0), left +3.0% (P = .003, d = 0.8)<br><br>- Confidence intervals: Not mentioned<br><br>- Statistical significance (p-values):<br><br>- Chi-square test: P = .006<br><br>- Injuries during training: P < .0001	- Blinding procedures: Not mentioned<br><br>- Potential sources of bias: Not mentioned<br><br>- Completeness of follow-up: Not mentioned<br><br>- Conflicts of interest: Not mentioned

								- Injuries during matches: P = .006<br><br>- Epidemiologic incidence: P = .023<br><br>- Incidence rate: P = .012<br><br>- CMJ improvement: P < .0001<br><br>- YBT improvement: right P = .001, left P = .003<br><br>- Relative risk or other comparative metrics: Not mentioned	
Neuromuscular training injury prevention strategies in youth sport: a systematic review and meta-analysis	C. Emery, Thierry-Olivier Roy, J. L. Whittaker, A. Nettel-Aguirre, W. van Mechelen	2015	Systematic review, Meta-analysis	Not mentioned (the abstract does not provide detailed information about participant characteristics such as total sample size, age range, gender distribution, or specific inclusion/exclusion criteria)	- Precise type of intervention: Neuromuscular training<br><br>- Duration of intervention: Not mentioned<br><br>- Frequency of intervention: Not mentioned<br><br>- Specific protocols or techniques used: Not mentioned	Not mentioned (the abstract does not provide specific details about the control conditions)	- Specific outcomes measured: Lower extremity and knee injury outcomes<br><br>- Measurement tools or methods: Incidence rate ratio (IRR)<br><br>- Timing of outcome measurements: During sport participation	- Lower extremity injury: IRR=0.64 (95% CI 0.49 to 0.84), statistically significant<br><br>- Knee injury: IRR=0.74 (95% CI 0.51 to 1.07), not statistically significant	- Randomization method: Not mentioned<br><br>- Blinding procedures: Not mentioned<br><br>- Potential sources of bias: Lack of uptake and ongoing maintenance of programs<br><br>- Completeness of follow-up: Not mentioned<br><br>- Conflicts of interest: Not mentioned
