# Supplementary material for: Exercise-Based Strategies from Warm-Up to Training: A Systematic Review of Performance Enhancement and Injury Prevention
Source: Sports (Basel). 2026 May 6;14(5):187. doi: 10.3390/sports14050187 (PMC13210987; doi:10.3390/sports14050187)
Supplement: Supplementary file 1 [file sports-14-00187-s001.zip › Supplementary Table S1b.pdf]

Supplementary Table S1b. CSV-derived dataset (reduced columns) — Eccentric / Nordic Hamstring.

Displayed columns: Title; Authors; Year; Study Design; Participant Characteristics; Intervention Type and Characteristics; Comparison/Control Conditions; Primary Outcome Measures; Key Findings and Statistical Results; Risk of Bias Assessment

Title	Authors	Year	Study Design	Participant Characteristics	Intervention Type and Characteristics	Comparison/Control Conditions	Primary Outcome Measures	Key Findings and Statistical Results	Risk of Bias Assessment
The Effects of Nordic Hamstring Exercise on Performance and Injury in the Lower Extremities: An Umbrella Review	Hugo Nunes, Luís Gonçalves Fernandes, Pedro Nunes Martins, Ricardo Maia Ferreira	2024	Systematic review (umbrella review)	- Total sample size: 17,260<br><br>- Age range or mean age: Not mentioned<br><br>- Gender distribution: Not mentioned<br><br>- Population type: Injured and non-injured athletes, recreationally active or healthy individuals<br><br>- Specific inclusion/exclusion criteria: Not mentioned	- Precise type of intervention: Nordic Hamstring Exercise (NHE), an eccentric exercise<br><br>- Duration of intervention: Not specified<br><br>- Frequency of intervention: Targeting 48 reps/week<br><br>- Specific protocols or techniques used: Nordic Hamstring Exercise; integration into warm-up phase	- Type of control: No intervention, placebo, or other interventions<br><br>- Specific details of control condition: Not specified<br><br>- How control condition differs from intervention group: Not specified	- Specific outcomes measured: sprint performance, muscle activation, eccentric strength, muscle architecture (fascicle length, muscle thickness, and pennation angle), hamstring injury prevention (up to 51%)<br><br>- Measurement tools or methods: Not mentioned<br><br>- Timing of outcome measurements: Not mentioned	Not mentioned (the abstract does not provide specific statistical results, effect sizes, confidence intervals, or p-values for muscle performance or injury prevention)	Not mentioned (the abstract does not provide explicit information on randomization methods, blinding procedures, potential sources of bias, completeness of follow-up, or conflicts of interest)
Specific interventions for prevention of muscle injury in lower limbs: systematic review and meta-analysis	J. Lopes, A. F. Machado, Allysiê Priscila Cavina, Jéssica Kirsch Michelletti, A. C. Almeida, C. M. Pastre	2019	Systematic review, Meta-analysis	Not mentioned (the abstract does not provide specific details about participant characteristics such as total sample size, age range, gender distribution, or specific inclusion/exclusion criteria)	- Warm-up: No specific details on duration, frequency, or protocols.<br><br>- Neuromuscular training: No specific details on duration, frequency, or protocols.<br><br>- Eccentric exercise: No specific details on duration,	Not mentioned (the abstract does not specify the control or comparison conditions)	- Specific outcomes measured: Incidence of muscle injuries<br><br>- Measurement tools or methods: Risk Ratio (RR) with 95% confidence interval<br><br>- Timing of outcome measurements: Not explicitly mentioned	- Eccentric exercise: RR = 3.49, 95% CI = 2.36 to 5.16, $p < 0.00001$<br><br>- Neuromuscular training: RR = 2.73, 95% CI = 2.03 to 2.68, $p < 0.00001$<br><br>- Warm-up: RR = 1.57, 95% CI = 0.92 to 2.7, p = 0.10	Not mentioned (the abstract does not provide information on the risk of bias assessment, randomization, blinding, potential sources of bias, completeness of follow-up, or conflicts of interest)

					frequency, or protocols.				
Evidence-Based Hamstring Injury Prevention and Risk Factor Management: A Systematic Review and Meta-analysis of Randomized Controlled Trials	Samuel S. Rudisill, Nathan H. Varady, M. Kucharik, Christopher T. Eberlin, S. Martin	2022	Systematic review, Meta-analysis	- Population type: Recreational, semiprofessional, and professional adult athletes<br><br>- Not mentioned (no information on total sample size, age range or mean age, gender distribution, or specific inclusion/exclusion criteria)	- Precise type of intervention: Eccentric training, concentric training, blood flow-restricted training, whole-body vibration, heavy back squat, FIFA 11+, plyometric training, static stretching, proprioceptive neuromuscular facilitation, dynamic stretching, sprint training<br><br>- Duration of intervention: Not mentioned<br><br>- Frequency of intervention: Not mentioned<br><br>- Specific protocols or techniques used: Not mentioned	Not mentioned (the abstract does not specify the control or comparison conditions)	- Specific outcomes measured: Muscle injury incidence, concentric and eccentric hamstring strength, flexibility, fascicle length, hamstring/quadriceps ratio, limb strength asymmetry<br><br>- Measurement tools or methods: Quantitative assessments using mean differences and confidence intervals<br><br>- Timing of outcome measurements: Not explicitly mentioned	- Eccentric training reduces hamstring injury incidence by 56.8% to 70.0%.<br><br>- Concentric hamstring strength increases with eccentric training (MD, 14.29 N·m; 95% CI, 8.53-20.05 N·m).<br><br>- Eccentric strength benefits from eccentric training (MD, 26.94 N·m; 95% CI, 15.59-38.30 N·m).<br><br>- Static stretching produces greater flexibility gains (MD, 10.89°; 95% CI, 8.92°-12.86°).<br><br>- Fascicle length increases with eccentric training (MD, 0.90 cm; 95% CI, 0.53-1.27 cm).<br><br>- Functional H/Q ratio improves with eccentric training (MD, 0.10; 95% CI, 0.03-0.16).<br><br>- Eccentric training reduces limb strength asymmetry.	Not mentioned (the abstract does not provide explicit information on risk of bias assessment, randomization methods, blinding procedures, potential sources of bias, completeness of follow-up, or conflicts of interest)

Eccentric hamstring muscle training can prevent hamstring injuries in soccer players.	A. Schache	2012	Randomized controlled trial	- Total sample size: 942 players<br><br>- Age range or mean age: Not mentioned<br><br>- Gender distribution: Male<br><br>- Population type: First team squad soccer players from top 5 national soccer divisions<br><br>- Specific inclusion/exclusion criteria: Included - first team squad soccer players from top 5 national soccer divisions; Excluded - players who joined a team after the start of the trial	- Precise type of intervention: Eccentric hamstring muscle training<br><br>- Duration of intervention: 27 sessions over a 10-week period during the midseason break, and once a week in the second half of the season<br><br>- Frequency of intervention: 1-3 sessions per week<br><br>- Specific protocols or techniques used: The Nordic curl exercise; players completed 2-3 sets of 5-12 repetitions	- Type of control: No intervention<br><br>- Specific details of control condition: The control group followed their usual training program without any additional eccentric hamstring muscle training.<br><br>- How control condition differs from intervention group: The control group did not receive the eccentric hamstring muscle training sessions that the intervention group received.	- Specific outcomes measured: Number of overall, new, and recurrent acute hamstring injuries<br><br>- Measurement tools or methods: Counting of acute physical complaints in the posterior thigh region during soccer matches or training<br><br>- Timing of outcome measurements: During one full soccer season	- Primary statistical results: 15 hamstring injuries in the intervention group, 52 in the control group.<br><br>- Effect sizes: NNT to prevent 1 hamstring injury = 13 (95% CI 9 to 23), NNT to prevent 1 new injury = 25 (95% CI 15 to 72), NNT for recurrent injury = 3 (95% CI 2 to 6).<br><br>- Confidence intervals: 9 to 23 for any injury, 15 to 72 for new injuries, 2 to 6 for recurrent injuries.<br><br>- Statistical significance: Not explicitly mentioned, but implied by significant differences in injury rates.<br><br>- Relative risk or comparative metrics: Indicated by NNT values comparing intervention to control groups.	- Randomization method: Cluster randomization with concealed allocation.<br><br>- Blinding procedures: Not mentioned.<br><br>- Potential sources of bias: Not explicitly mentioned.<br><br>- Completeness of follow-up: High, with 50 teams and 942 players completing the study.<br><br>- Conflicts of interest: Not mentioned.
Strength training as superior, dose-dependent and safe prevention of acute and overuse sports injuries: a	Jeppe Bo Lauersen, T. Andersen, L. Andersen	2018	Systematic review, Meta-analysis	- Total sample size: 7738<br><br>- Age range: 12 to 40 years	- Coppack et al.: 14-week program, four daily strength training exercises, focusing on concentric and	Not mentioned (the paper does not provide specific details about the control conditions)	- Specific outcomes measured: Muscle injury incidence (e.g., hamstring muscle injuries, ACL knee injuries)	- Primary statistical results: Cluster-adjusted intention-to-treat analysis RR = 0.338 (95% CI 0.238 to 0.480,	- Randomization method: Not explicitly detailed, but cluster-randomization was used in some

systematic review, qualitative analysis and meta-analysis				- Gender distribution: Not explicitly detailed; includes male and female participants<br><br>- Population type: Military conscripts and soccer players (elite and amateur)<br><br>- Specific inclusion criteria: Free of injury at inclusion, randomized controlled trial, adequate volume and intensity of intervention, adequate follow-up period	eccentric contractions for hip and knee extensors.<br><br>- Waldén et al.: 20-minute, two sessions per week, balance, core stability, and knee alignment program.<br><br>- Zouita et al.: 36 sessions of 10 exercises over 90 minutes, loads from 30% to 80% 1RM, technique familiarization, individualized loads, and periodization.<br><br>- Compliance measures: coach instruction, supervision, written material, participant diaries.		- Measurement tools or methods: Relative risk (RR) estimate<br><br>- Timing of outcome measurements: Follow-up periods ranging from 14 weeks to one season (7 to 12 months)	P<0.0001)<br><br>- Effect sizes: A 10% increase in strength training volume reduces injury risk by more than four percentage points<br><br>- Confidence intervals: 95% CI for RR = 0.238 to 0.480; for injury reduction = 52% to 76%<br><br>- Statistical significance: P<0.0001 for RR; P<0.005 for volume and intensity in meta-regression<br><br>- Relative risk or other comparative metrics: RR = 0.338; average injury reduction = 66%	studies.<br><br>- Blinding procedures: Difficulties with participant and outcome assessor blinding were noted.<br><br>- Potential sources of bias: Language bias due to only including English manuscripts; some studies did not adjust for cluster-randomization.<br><br>- Completeness of follow-up: Low risk of attrition bias due to intention-to-treat analyses or no missing data.<br><br>- Conflicts of interest: No financial conflicts declared; language bias acknowledged.
Effects of a 10-week in-season eccentric-overload training program on muscle-injury prevention and performance in junior elite soccer players.	M. de Hoyo, M. Pozzo, B. Sañudo, Luis Carrasco, O. Gonzalo-Skok, Sergio Domínguez-Cobo, Eduardo Morán-Camacho	2015	Quasi-experimental study	- Total sample size: 36<br><br>- Age range: 17 to 19 years old<br><br>- Gender distribution: Not explicitly mentioned, likely male<br><br>- Population type: Junior elite soccer players	- Precise type of intervention: Eccentric-overload training program using flywheel ergometers for half-squat and leg-curl exercises.<br><br>- Duration of intervention: 10 weeks.<br><br>- Frequency of intervention: 1 or 2 sessions per week.	- Type of control: No intervention<br><br>- Specific details of control condition: Participants in the control group continued with their usual technical/tactical training without any additional strength training.<br><br>- How control condition differs	- Specific outcomes measured: Muscle injury incidence, injury severity, countermovement jump (CMJ), 10-m sprint, 20-m sprint<br><br>- Measurement tools or methods: OptoJump system for CMJ, timing for sprint tests, medical staff recording for injury incidence and severity	- Primary statistical results: Lower number of days of absence per injury and possible decrement in injury incidence per 1000 hours of match play in EXP compared to CON.<br><br>- Effect sizes: ES = 0.94 for days of absence, ES = 0.37 for 20-m sprint time, ES = 0.77 for	- Randomization method: Nonrandomized study design, which is a significant source of bias.<br><br>- Blinding procedures: Not mentioned, indicating potential bias in outcome assessment.<br><br>- Potential sources

				- Specific inclusion criteria: Part of a first Spanish soccer division club academy squad, no severe lower-limb muscle injuries in the previous two months<br><br>- Specific exclusion criteria: Severe lower-limb muscle injuries in the previous two months	- Specific protocols or techniques used:<br><br>- Half-squat exercise: Bending knees up to 90° during eccentric phase, then performing concentric phase as fast as possible.<br><br>- Leg-curl exercise: Bilateral knee flexion in prone position, accelerating and decelerating flywheel through concentric and eccentric actions.<br><br>- Volume increase: 3 sets of 6 repetitions in weeks 1-4, 4 sets in weeks 5-6, 5 sets in weeks 7-8, 6 sets in weeks 9-10.	from intervention group: The control group did not receive the eccentric-overload training program, which was the main intervention in the experimental group.	- Timing of outcome measurements: 1 week before and 1 week after the intervention, injury incidence and severity monitored throughout the season	10-m flying-sprint time, ES = 0.79 for CMJ.<br><br>- Confidence intervals: 90%CL for injury severity = 10.1; 86.4, 90%CL for incidence per 1000 hours of match play = -115.0; 72.9.<br><br>- Statistical significance: Substantial improvements in CMJ, 20-m sprint time, and 10-m flying-sprint time in EXP.<br><br>- Relative risk or comparative metrics: Lower injury severity and incidence in EXP compared to CON.	of bias: Nonrandomized design, lack of comprehensive strength measurements, and limited performance tests.<br><br>- Completeness of follow-up: Participants not attending 80% of sessions were omitted, which could introduce bias.<br><br>- Conflicts of interest: Not mentioned.
The Effects of Pre-conditioning on Exercise-Induced Muscle Damage: A Systematic Review and Meta-analysis	Lachlan Boyd, G. Deakin, Baily Devantier-Thomas, Utkarsh Singh, K. Doma	2023	Systematic review, Meta-analysis	- Total sample size: Pre-conditioning groups = 427 participants, Control groups = 261 participants<br><br>- Age range or mean age: Pre-conditioning groups = 20.3 years (21-70 years), Control groups = 22.1 years (20-30 years)<br><br>- Gender	- Precise type of intervention: Low-intensity eccentric contractions, short-duration maximal voluntary contractions (MVCs), downhill walking<br><br>- Duration of intervention: Single bout, minimum of 24 hours before damaging exercise<br><br>- Frequency of	- Type of control: No intervention (no pre-conditioning)<br><br>- Specific details of control condition: Control groups did not perform pre-conditioning exercises before the muscle-damaging protocol. In some studies, the control group was the group that performed equivalent exercises without pre-	- Specific outcomes measured: Creatine kinase (CK), myoglobin, maximal voluntary contraction (MVC), maximal isometric torque, peak isometric force, peak eccentric force, peak concentric force, maximal eccentric contraction, muscle soreness, swelling, degrees of joint motion.<br><br>- Measurement tools	- Creatine kinase levels: Lower in pre-conditioning group at 24 h (SMD = -1.64; Z = 8.39; p = 0.00001), 48 h (SMD = -2.65; Z = 7.78; p = 0.00001), 72 h (SMD = -2.39; Z = 5.71; p = 0.00001), and 96 h (SMD = -3.52; Z = 7.39; p = 0.00001).<br><br>- Delayed-onset muscle soreness: Lower in pre-conditioning group	- Randomization method: Not explicitly mentioned<br><br>- Blinding procedures: Not explicitly mentioned<br><br>- Potential sources of bias: High inter-study heterogeneity, publication bias, lack of specificity in subject selection,

				distribution: Not specified<br><br>- Population type: General population or 'untrained' participants<br><br>- Specific inclusion/exclusion criteria: No resistance training experiences in the previous 6 or more months	intervention: Single bout<br><br>- Specific protocols or techniques used: Low-intensity eccentric contractions at 10-40% of maximum effort, short-duration MVCs repeated several times, downhill walking for 5 minutes	conditioning.<br><br>- How control condition differs from intervention group: The control group did not receive any pre-conditioning exercises, unlike the intervention group which received pre-conditioning exercises before the muscle-damaging protocol.	or methods: Visual analogue scales for muscle soreness, girth circumference for swelling, percentage change in degrees for joint motion.<br><br>- Timing of outcome measurements: 24 hours, 48 hours, 72 hours, 96 hours post-exercise.	at 24 h (SMD = -1.89; Z = 6.17; p = 0.00001), 48 h (SMD = -2.50; Z = 7.99; p = 0.00001), 72 h (SMD = -2.73; Z = 7.86; p = 0.00001), and 96 h (SMD = -3.30; Z = 8.47; p = 0.00001).<br><br>- Maximal voluntary contraction force: Better maintained in pre-conditioning group at 24 h (SMD = 1.46; Z = 5.49; p = 0.00001), 48 h (SMD = 1.59; Z = 6.04; p = 0.00001), 72 h (SMD = 2.02; Z = 6.09; p = 0.00001), and 96 h (SMD = 2.16; Z = 5.69; p = 0.00001).<br><br>- Range of motion: Better maintained in pre-conditioning group at 24 h (SMD = 1.48; Z = 4.30; p = 0.00001), 48 h (SMD = 2.20; Z = 5.64; p = 0.00001), 72 h (SMD = 2.66; Z = 5.42; p = 0.00001), and 96 h (SMD = 2.5; Z = 5.46; p = 0.00001).<br><br>- Certainty of evidence: Very low due to limited sample size, publication bias, and high inter-study heterogeneity.	absence of washout periods<br><br>- Completeness of follow-up: Not explicitly mentioned<br><br>- Conflicts of interest: Authors have no conflicts of interest

EFFECTIVE EXERCISE BASED TRAINING INTERVENTIONS TARGETING INJURY PREVENTION IN TEAM-BASED SPORTS: A SYSTEMATIC REVIEW	E. O'Malley, J. Murphy, C. Gissane, U. McCarthy-Persson, Catherine Blake	2014	Systematic review, Meta-analysis	- Total sample size: 21,479<br><br>- Age range or mean age: Not mentioned<br><br>- Gender distribution: Not mentioned<br><br>- Population type: Athletes or individuals involved in team sports<br><br>- Specific inclusion/exclusion criteria: Not mentioned	- Multifaceted exercise programs<br><br>- Balance board training<br><br>- Hamstring strength training<br><br>- Eccentric training of the Achilles and patellar tendons<br><br>- No specific details on duration, frequency, or protocols provided	- Type of control: Normal practice (no specific intervention)<br><br>- Specific details of control condition: Normal practice or usual routines<br><br>- How control condition differs from intervention group: Control group followed normal practice without additional exercise programs aimed at injury prevention	- Specific outcomes measured: Overall injury incidence, lower-limb injury incidence, knee injury risk, ACL injury risk, ankle injury risk, hamstring injury risk<br><br>- Measurement tools or methods: Risk ratios (RR) and 95% confidence intervals (95% CI) calculated using the Mantel-Haenszel method in a random effects model<br><br>- Timing of outcome measurements: Not mentioned	- Multifaceted exercise programs: Overall injury risk RR=0.65, 95% CI=0.44–0.96, P=.03; Knee injury risk RR=0.79, 95% CI=0.63–0.99, P=.04; ACL injury risk RR=0.51, 95% CI=0.28–0.93, P=.03; Ankle injury risk RR=0.72, 95% CI=0.58–0.90, P=.003.<br><br>- Balance board interventions: Hamstring injury risk RR=0.22, 95% CI=0.05–1.02, P=.05; Ankle injury risk RR=0.64, 95% CI=0.49–0.83, P=.001.<br><br>- Hamstring muscle training: No significant effect, RR=0.46, 95% CI=0.19–1.11, P=.09.<br><br>- Eccentric training of Achilles and patellar tendons: Increased injury risk, RR=2.5, 95% CI=1.35- 4.61, P=.003.	Not mentioned (the abstract does not provide specific details on randomization method, blinding procedures, potential sources of bias, completeness of follow-up, or conflicts of interest)
The effectiveness of a neuromuscular prevention strategy to reduce injuries in youth soccer: a cluster-randomised controlled trial	C. Emery, W. Meeuwisse	2010	Randomized controlled trial	- Total sample size: 744 players (380 training group, 364 control group)<br><br>- Age range: Adolescents between the ages of	- Precise type of intervention: Soccer-specific neuromuscular training programme including dynamic stretching, eccentric	- Type of control: Alternative intervention<br><br>- Specific details of control condition: Standardised warm-up (static and	- Specific outcomes measured: Injury rate (injuries/1000 player-hours)<br><br>- Measurement tools or methods: Injury report forms (IRFs),	- Injury rates: Training group = 2.08 injuries/1000 player-hours, Control group = 3.35 injuries/1000 player-hours	- Randomization method: Cluster-randomized controlled trial with allocation concealment.<br><br>- Blinding

				13 and 18<br><br>- Gender distribution:<br><br>- Training group: Female=42.4%, Male=57.6%<br><br>- Control group: Female=69%, Male=31%<br><br>- Population type: Youth soccer players from Calgary soccer clubs (male or female, U13–U18, tier 1–2, indoor soccer)<br><br>- Inclusion criteria: Adolescents between 13 and 18 years old, participating in a Calgary Minor Soccer Association Club team<br><br>- Exclusion criteria: Injury within 6 weeks, systemic disease, or neurological disorder preventing full participation	strength, agility, jumping, and balance.<br><br>- Duration of intervention: 15 minutes for warm-up (5 minutes aerobic and dynamic stretching, 10 minutes neuromuscular training), additional 15 minutes for home-based balance training.<br><br>- Frequency of intervention: At least three times per week.<br><br>- Specific protocols or techniques: Use of a wobble board for balance training, components selected from previously used programmes in youth sports.	dynamic stretching and aerobic components), home-based stretching program<br><br>- How control condition differs from intervention group: Does not include neuromuscular training components or home-based balance training with a wobble board	assessment by a study therapist<br><br>- Timing of outcome measurements: During the 20-week soccer season (October 2006-March 2007), with follow-up until October 2007	- Incidence Rate Ratios (IRR): All injuries = 0.62 (95% CI 0.39 to 0.99), Acute onset injuries = 0.57 (95% CI 0.35 to 0.91)<br><br>- Statistical significance: p=0.045 for all injuries, p=0.018 for acute onset injuries<br><br>- Relative risk reduction: Training group had a lower risk of all injuries and acute onset injuries compared to the control group	procedures: Study therapist blinded to group allocation.<br><br>- Potential sources of bias: Differential non-participation rates, imputation of exposure data, low return rate of home-programme journals.<br><br>- Completeness of follow-up: Imputation of weekly exposure data for some teams; low return rate of home-programme journals.<br><br>- Conflicts of interest: Financial support from Fitter International for wobble boards.
