# Supplementary material for: Exercise-Based Strategies from Warm-Up to Training: A Systematic Review of Performance Enhancement and Injury Prevention
Source: Sports (Basel). 2026 May 6;14(5):187. doi: 10.3390/sports14050187 (PMC13210987; doi:10.3390/sports14050187)
Supplement: Supplementary file 1 [file sports-14-00187-s001.zip › Supplementary Table S1c.pdf]

Supplementary Table S1c. CSV-derived dataset (reduced columns) — Strength Training.

Displayed columns: Title; Authors; Year; Study Design; Participant Characteristics; Intervention Type and Characteristics; Comparison/Control Conditions; Primary Outcome Measures; Key Findings and Statistical Results; Risk of Bias Assessment

Title	Authors	Year	Study Design	Participant Characteristics	Intervention Type and Characteristics	Comparison/Control Conditions	Primary Outcome Measures	Key Findings and Statistical Results	Risk of Bias Assessment
The effectiveness of neuromuscular warm-up strategies, that require no additional equipment, for preventing lower limb injuries during sports participation: a systematic review	Katherine Herman, C. Barton, P. Malliaras, D. Morrissey	2012	Systematic review	- Total sample size: Average of 1,500 participants (range 1,020 to 2,020)<br><br>- Age range or mean age: 13 to 26 years<br><br>- Gender distribution: Two studies included male and female participants; seven studies included female participants only<br><br>- Population type: Amateur football players, army recruits, amateur football and basketball players, amateur football, basketball, and volleyball players<br><br>- Specific inclusion/exclusion criteria: Studies must investigate neuromuscular warm-up strategies without additional equipment, focus on preventing lower limb injuries, use functional training, and be easily incorporated	- "The 11+": Duration - 8 months; Frequency - Not specified; Protocols - Stretching, strengthening, balance exercises, sports-specific agility drills, landing techniques.<br><br>- "KIPP": Duration - 1 year; Frequency - Not specified; Protocols - Stretching, strengthening, balance exercises, sports-specific agility drills, landing techniques.<br><br>- "PEP": Duration - 2 years; Frequency - Not specified; Protocols - Stretching, strengthening, balance exercises, sports-specific agility drills, landing techniques.<br><br>- "HarmoKnee": Duration - 9 months; Frequency - Not specified; Protocols - Stretching, strengthening,	The control conditions in the studies reviewed by Herman et al. (2012) involved participants performing their usual warm-up routines, which did not include the specific neuromuscular exercises being tested in the intervention groups. These control conditions served as a baseline for comparison to assess the effectiveness of the intervention strategies in reducing lower limb injuries. The control groups differed from the intervention groups by not incorporating exercises such as stretching, strengthening, balance exercises, sports-specific agility drills, and landing techniques.	- Specific outcomes measured: Incidence of lower limb injuries (overall, knee, ankle)<br><br>- Measurement tools or methods: Risk ratios (RR) and confidence intervals (CI) calculated using Review Manager version 5.0<br><br>- Timing of outcome measurements: Varies across studies (12 weeks to two years)	- "The 11+" strategy: RR 0.67 (CI 0.54 to 0.84) for overall lower limb injuries, RR 0.45 (CI 0.28 to 0.71) for overuse injuries, RR 0.48 (CI 0.32 to 0.72) for knee injuries.<br><br>- KIPP strategy: RR 0.5 (CI 0.33 to 0.76) for non-contact lower limb injuries, RR 0.44 (CI 0.22 to 0.86) for overuse injuries.<br><br>- PEP strategy: RR 0.18 (CI 0.08 to 0.42) for ACL injuries.<br><br>- HarmoKnee program: RR 0.22 (CI 0.06 to 0.76) for knee injuries.<br><br>- AKP PTP: RR 0.27 (CI 0.14 to 0.54) for anterior knee pain.<br><br>- NNT values: "11+" (18), KIPP (24), HarmoKnee (72), PEP (70), AKP PTP (28).	- Randomization method: Inadequate or absent randomization in some studies.<br><br>- Blinding procedures: Failure to blind participants and researchers in some studies.<br><br>- Potential sources of bias: High drop-out rates, poor compliance, different group values at baseline.<br><br>- Completeness of follow-up: Not explicitly mentioned, but high drop-out rates indicate potential issues.<br><br>- Conflicts of interest: Authors declare no competing interests.

				into regular activity. Excluded studies using home-based exercises, equipment like wobble boards, or those not part of a warm-up program.	balance exercises, sports-specific agility drills, landing techniques.<br><br>- "AKP PTP": Duration - 14 weeks; Frequency - 7 times a week, 105 minutes; Protocols - Stretching, strengthening, balance exercises, sports-specific agility drills, landing techniques.				
