# Supplementary material for: Exercise-Based Strategies from Warm-Up to Training: A Systematic Review of Performance Enhancement and Injury Prevention
Source: Sports (Basel). 2026 May 6;14(5):187. doi: 10.3390/sports14050187 (PMC13210987; doi:10.3390/sports14050187)
Supplement: Supplementary file 1 [file sports-14-00187-s001.zip › Supplementary Table S1d.pdf]

Supplementary Table S1d. CSV-derived dataset (reduced columns) — Multicomponent / Policy / Program.

Displayed columns: Title; Authors; Year; Study Design; Participant Characteristics; Intervention Type and Characteristics; Comparison/Control Conditions; Primary Outcome Measures; Key Findings and Statistical Results; Risk of Bias Assessment

Title	Authors		Year	Study Design	Participant Characteristics	Intervention Type and Characteristics	Comparison/Control Conditions	Primary Outcome Measures	Key Findings and Statistical Results	Risk of Bias Assessment
Prevention of severe knee injuries in men's elite football by implementing specific training modules	W. Krutsch, J. Lehmann, P. Jansen, P. Angele, Birgit Fellner, Leonard Achenbach, V. Krutsch, M. Nerlich, V. Alt, O. Loose		2019	Cohort study	- Total sample size: Study group - 529 players, Control group - 601 players<br><br>- Age range or mean age: Not mentioned<br><br>- Gender distribution: Male (inferred from "men's elite football")<br><br>- Population type: Elite football players<br><br>- Specific inclusion/exclusion criteria: Not mentioned	- Precise type of intervention: Injury prevention programme with 5 modules<br><br>- Specific protocols or techniques used: Postural stability, mobilisation of lower extremity joints, leg and trunk stabilisation, jumping, and landing exercises as well as agility movements<br><br>- Duration of intervention: Not mentioned<br><br>- Frequency of intervention: Not mentioned	- Type of control: Alternative intervention (standard training program)<br><br>- Specific details of control condition: Teams continued their standard training program<br><br>- How control condition differs from intervention group: Control group did not use the new, specifically adapted training modules	- Specific outcomes measured: Reduction in severe knee injuries<br><br>- Measurement tools or methods: Injury report over one season<br><br>- Timing of outcome measurements: Over one season	- Primary statistical results: Incidence of severe knee injuries in the study group: 0.38 per 1000 hours; Control group: 0.68 per 1000 hours. Prevalence in study group: 9.8%; Control group: 18.0%.<br><br>- Effect sizes: Not explicitly mentioned.<br><br>- Confidence intervals: Not mentioned.<br><br>- Statistical significance: $p < 0.05$.<br><br>- Relative risk or comparative metrics: Study group had a lower incidence of severe knee injuries compared to the control group.	- Randomization method: Not mentioned<br><br>- Blinding procedures: Not mentioned<br><br>- Potential sources of bias: Not explicitly addressed<br><br>- Completeness of follow-up: Not mentioned<br><br>- Conflicts of interest: Not mentioned
