# Supplementary material for: Exercise-Based Strategies from Warm-Up to Training: A Systematic Review of Performance Enhancement and Injury Prevention
Source: Sports (Basel). 2026 May 6;14(5):187. doi: 10.3390/sports14050187 (PMC13210987; doi:10.3390/sports14050187)
Supplement: Supplementary file 1 [file sports-14-00187-s001.zip › Supplementary Table S1e.pdf]

Supplementary Table S1e. CSV-derived dataset (reduced columns) — Load Monitoring / Quantification.

Displayed columns: Title; Authors; Year; Study Design; Participant Characteristics; Intervention Type and Characteristics; Comparison/Control Conditions; Primary Outcome Measures; Key Findings and Statistical Results; Risk of Bias Assessment

Title	Authors	Year	Study Design	Participant Characteristics	Intervention Type and Characteristics	Comparison/Control Conditions	Primary Outcome Measures	Key Findings and Statistical Results	Risk of Bias Assessment
The Use of Acute Exercise Interventions as Game Day Priming Strategies to Improve Physical Performance and Athlete Readiness in Team-Sport Athletes: A Systematic Review	Billy Mason, A. Mckune, K. Pumpa, N. Ball	2020	Systematic review	- Population type: Team-sport athletes<br><br>- Other participant characteristics: Not mentioned	- Precise type of intervention: Resistance training, cycling, running<br><br>- Duration of intervention: Implemented 1-12 hours prior to competition<br><br>- Frequency of intervention: Not mentioned<br><br>- Specific protocols or techniques used: Resistance training using heavy loads at low volumes; running-based sprint priming	Not mentioned (the abstract does not provide specific details about the control conditions)	- Specific outcomes measured: strength and power measures, sprint and repeat sprint performance, counter-movement jump height<br><br>- Measurement tools or methods: Not specified<br><br>- Timing of outcome measurements: 1-12 hours after exercise intervention; specific time points include 4-6 hours for resistance training and 5-6 hours for running-based sprint priming	Not mentioned (the abstract does not provide specific statistical results, effect sizes, confidence intervals, or p-values)	- Randomization method: Not mentioned<br><br>- Blinding procedures: Not mentioned<br><br>- Potential sources of bias: Exclusion criteria suggest efforts to control for certain biases<br><br>- Completeness of follow-up: Not mentioned<br><br>- Conflicts of interest: Not mentioned
