# Supplementary material for: Exercise-Based Strategies from Warm-Up to Training: A Systematic Review of Performance Enhancement and Injury Prevention
Source: Sports (Basel). 2026 May 6;14(5):187. doi: 10.3390/sports14050187 (PMC13210987; doi:10.3390/sports14050187)
Supplement: Supplementary file 1 [file sports-14-00187-s001.zip › Supplementary Table S1f.pdf]

Supplementary Table S1f. CSV-derived dataset (reduced columns) — Preseason Ramp-up.

Displayed columns: Title; Authors; Year; Study Design; Participant Characteristics; Intervention Type and Characteristics; Comparison/Control Conditions; Primary Outcome Measures; Key Findings and Statistical Results; Risk of Bias Assessment

Title	Authors	Year	Study Design	Participant Characteristics	Intervention Type and Characteristics	Comparison/Control Conditions	Primary Outcome Measures	Key Findings and Statistical Results	Risk of Bias Assessment
882 FO33 – Bringing athletes back to sport safely: data-driven gradual practice ramp-up strategy during preseason reduces lower extremity strains in U.S. national football league	Mackenzie Herzog, Kristin Shiue, Rebecca Y. Lee, Leigh Weiss, Kristy B. Arbogast, Tyler Williams, Allen Sills, Christina Mack	2024	Observational study	- Total sample size: Not mentioned (implied to be all NFL players participating during 2018–2022)<br><br>- Age range or mean age: Not mentioned<br><br>- Gender distribution: Not mentioned (likely majority male)<br><br>- Population type: Athletes (NFL players)<br><br>- Specific inclusion/exclusion criteria: Not mentioned	- Precise type of intervention: Gradual 15-minute ramp-up<br><br>- Duration of intervention: 15-minute ramp-up<br><br>- Frequency of intervention: Not mentioned<br><br>- Specific protocols or techniques used: Mandating maximum practice durations, individual Club education sessions	- Type of control: Alternative intervention<br><br>- Specific details of control condition: <10-minute ramp-up, high-low duration variation, stable duration, and reverse ramp-up<br><br>- How control condition differs from intervention group: These strategies are compared to the gradual 15-minute ramp-up strategy in terms of LEX strain rates.	- Specific outcomes measured: Strains to the hamstring, quadriceps, adductor, and calf<br><br>- Measurement tools or methods: Incidence rates per 100 practice-minutes and 95% confidence intervals<br><br>- Timing of outcome measurements: During and following Training Camp; pre-intervention in 2021, post-intervention in 2022	- Primary statistical results: League-wide LEX strain incidence during Training Camp decreased by 25% from 286 in 2021 to 215 in 2022.<br><br>- Effect sizes: Not mentioned.<br><br>- Confidence intervals: Not mentioned.<br><br>- Statistical significance (p-values): Not mentioned.<br><br>- Relative risk or other comparative metrics: Clubs using the gradual 15-minute ramp-up strategy had lower LEX strain rates compared to other strategies, particularly the reverse ramp-up strategy.	- Randomization method: Not applicable (observational study)<br><br>- Blinding procedures: Not mentioned<br><br>- Potential sources of bias: Observational study design, lack of control group<br><br>- Completeness of follow-up: Not mentioned<br><br>- Conflicts of interest: Not mentioned
