# Supplementary material for: Exercise-Based Strategies from Warm-Up to Training: A Systematic Review of Performance Enhancement and Injury Prevention
Source: Sports (Basel). 2026 May 6;14(5):187. doi: 10.3390/sports14050187 (PMC13210987; doi:10.3390/sports14050187)
Supplement: Supplementary file 1 [file sports-14-00187-s001.zip › Supplementary Table S1g.pdf]

Supplementary Table S1g. CSV-derived dataset (reduced columns) — Other / Unspecified.

Displayed columns: Title; Authors; Year; Study Design; Participant Characteristics; Intervention Type and Characteristics; Comparison/Control Conditions; Primary Outcome Measures; Key Findings and Statistical Results; Risk of Bias Assessment

Title	Authors	Year	Study Design	Participant Characteristics	Intervention Type and Characteristics	Comparison/Control Conditions	Primary Outcome Measures	Key Findings and Statistical Results	Risk of Bias Assessment
Prevention of physical training-related injuries recommendations for the military and other active populations based on expedited systematic reviews.	S. Bullock, B. Jones, J. Gilchrist, S. Marshall	2010	Systematic review	Not mentioned (the abstract does not provide specific participant characteristics such as total sample size, age range, gender distribution, or specific inclusion/exclusion criteria)	- Prevent overtraining: No specific details on duration, frequency, or protocols.<br><br>- Agility-like training: No specific details on duration, frequency, or protocols.<br><br>- Mouthguards: No specific details on duration, frequency, or protocols.<br><br>- Semirigid ankle braces: No specific details on duration, frequency, or protocols.<br><br>- Nutrient replacement: No specific details on duration, frequency, or protocols.<br><br>- Synthetic socks: No specific details on duration, frequency, or protocols.	Not mentioned (the abstract does not provide information on control or comparison conditions)	Not mentioned (the abstract does not provide specific primary outcome measures related to muscle performance and injury prevention)	Not mentioned (the abstract does not provide specific statistical results, effect sizes, confidence intervals, or statistical significance related to muscle performance or injury prevention)	Not mentioned (the abstract does not provide explicit information on risk of bias assessment, randomization, blinding, potential sources of bias, completeness of follow-up, or conflicts of interest)

Effect of an Injury Prevention Program on Muscle Injuries in Elite Professional Soccer	A. Owen, D. Wong, A. Dellal, D. Paul, E. Orhant, S. Collie	2013	Quasi-experimental study	- Total sample size: 26 (intervention season), 23 (control season)<br><br>- Age range or mean age: Not mentioned<br><br>- Gender distribution: Male<br><br>- Population type: Elite male professional soccer players<br><br>- Specific inclusion/exclusion criteria: Not mentioned	- Precise type of intervention: Not mentioned<br><br>- Duration of intervention: For the entirety of the season<br><br>- Frequency of intervention: Twice weekly<br><br>- Specific protocols or techniques used: Not mentioned	- Type of control: No intervention<br><br>- Specific details of control condition: The second season (2009–2010) served as the control season where no structured injury prevention program was implemented.<br><br>- How control condition differs from intervention group: The control season did not include the structured injury prevention program that was performed twice weekly during the intervention season.	- Specific outcomes measured: Number of muscle injuries, total number of injuries<br><br>- Measurement tools or methods: Not mentioned<br><br>- Timing of outcome measurements: Over two consecutive seasons (2008-2009 intervention season, 2009-2010 control season)	- Primary statistical results: Total injuries increased from 72 to 88; contusion injuries increased from 23 to 44.<br><br>- Effect sizes: Moderate effect for muscle injuries; large effect for squad size.<br><br>- Statistical significance: $p < 0.001$ for squad size effect.<br><br>- Relative risk or other comparative metrics: Not mentioned.	- Randomization method: Not mentioned (quasi-experimental design without randomization)<br><br>- Blinding procedures: Not mentioned<br><br>- Potential sources of bias: Small sample size, lack of randomization, no mention of blinding or conflicts of interest<br><br>- Completeness of follow-up: Not mentioned<br><br>- Conflicts of interest: Not mentioned
The Effect of Training Loads on Performance Measures and Injury Characteristics in Rugby League Players: A Systematic Review.	Mark Booth, R. Orr, S. Cobley	2017	Systematic review	Not mentioned (the abstract does not provide detailed information about participant characteristics such as total sample size, age range, gender distribution, or specific inclusion/exclusion criteria)	Not mentioned (the abstract does not provide specific details about the type, duration, frequency, or protocols of the interventions)	Not mentioned (the abstract does not provide information on control or comparison conditions)	- Specific outcomes measured: maximal aerobic power, vertical jump (in cm), agility scores (in s), muscular strains and joint sprains, lower-limb injuries, overexertion and overuse injuries<br><br>- Measurement tools or methods: session rating of perceived exertion<br><br>- Timing of outcome measurements:	Not mentioned (the abstract does not provide specific statistical results, effect sizes, confidence intervals, statistical significance, or relative risk metrics)	Not mentioned (the abstract does not provide explicit information on risk of bias assessment, randomization, blinding, potential sources of bias, completeness of follow-up, or conflicts of interest)

							preseason training phases		
