# Supplementary material for: Exercise-Based Strategies from Warm-Up to Training: A Systematic Review of Performance Enhancement and Injury Prevention
Source: Sports (Basel). 2026 May 6;14(5):187. doi: 10.3390/sports14050187 (PMC13210987; doi:10.3390/sports14050187)
Supplement: Supplementary file 1 [file sports-14-00187-s001.zip › Supplementary Table S2.pdf]

Supplementary Table S2

Risk of Bias and Methodological Quality Assessment of Included Studies

Study	Design	Tool Applied	Overall Judgment
**de Hoyo et al., 2015**	RCT	RoB 2	Low risk
**Lauersen et al., 2018**	Meta-analysis	AMSTAR 2	High confidence
**Faude et al., 2017**	Systematic Review & Meta-analysis	AMSTAR 2	High confidence
**Grooms et al., 2013**	RCT	RoB 2	Low risk
**Emery & Meeuwisse, 2010**	Cluster RCT	RoB 2	Low risk
**Rudisill et al., 2022**	Systematic Review & Meta-analysis	AMSTAR 2	High confidence
**Richmond et al., 2017**	Cluster RCT	RoB 2	Low risk
**Mason et al., 2020**	Systematic Review	AMSTAR 2	Moderate confidence
**Steib et al., 2017**	Meta-analysis	AMSTAR 2	High confidence
**Trecroci et al., 2020**	Controlled Study	ROBINS-I	Moderate risk
**Booth et al., 2017**	Systematic Review	AMSTAR 2	Moderate confidence
**Nunes et al., 2024**	Umbrella Review	AMSTAR 2	High confidence
**Paravlic et al., 2024**	Cluster RCT	RoB 2	Low risk
**O'Malley et al., 2014**	Systematic Review	AMSTAR 2	Moderate confidence
**Vlachas & Paraskevopoulos, 2022**	Systematic Review & Meta-analysis	AMSTAR 2	Moderate confidence
**Boyd et al., 2023**	Systematic Review & Meta-analysis	AMSTAR 2	High confidence
**Li & Zhu, 2025**	Systematic Review & Meta-analysis	AMSTAR 2	High confidence
**Stojanović et al., 2022**	Cluster RCT	RoB 2	Low risk
**Bonato et al., 2018**	Cluster RCT	RoB 2	Low risk
**Bustos et al., 2020**	Controlled Study	ROBINS-I	Moderate risk
**Emery et al., 2019**	Cluster RCT	RoB 2	Low risk
**Hilska et al., 2021**	Cluster RCT	RoB 2	Low risk
**Krutsch et al., 2019**	Controlled Study	ROBINS-I	Moderate risk
**Bullock et al., 2025**	Systematic Review & Meta-analysis	AMSTAR 2	High confidence
**Schache, 2012**	RCT	RoB 2	Low risk
**Dolan et al., 2023**	Controlled Study	ROBINS-I	Moderate risk
**Richmond et al., 2011**	Cluster RCT	RoB 2	Low risk
**Rahlf & Zech, 2019**	RCT	RoB 2	Some concerns
**Emery et al., 2018**	Cluster RCT	RoB 2	Some concerns
**Herzog et al., 2024**	Observational	ROBINS-I	Moderate risk
**Emery et al., 2020**	Cluster RCT	RoB 2	Low risk

**Berg et al., 2021**	Cluster RCT	RoB 2	Low risk
**Lopes et al., 2019**	Systematic Review & Meta-analysis	AMSTAR 2	Moderate confidence
**Behm et al., 2016**	Systematic Review	AMSTAR 2	High confidence
**Herman et al., 2012**	Systematic Review	AMSTAR 2	Moderate confidence
**Hübscher et al., 2010**	Systematic Review	AMSTAR 2	Moderate confidence
**Emery et al., 2015**	Systematic Review & Meta-analysis	AMSTAR 2	High confidence
**Owen et al., 2013**	Controlled Study	ROBINS-I	Moderate risk
**Bullock et al., 2010**	Systematic Review	AMSTAR 2	Moderate confidence
**Jafari et al., 2024**	Controlled Study	ROBINS-I	Moderate risk

Note: Randomized and cluster-randomized trials were assessed using the Cochrane RoB 2 tool. Non-randomized studies were evaluated using ROBINS-I. Systematic reviews and meta-analyses were assessed using AMSTAR 2. Overall judgments were incorporated qualitatively into the interpretation of findings.
