# Supplementary material for: Exercise-Based Strategies from Warm-Up to Training: A Systematic Review of Performance Enhancement and Injury Prevention
Source: Sports (Basel). 2026 May 6;14(5):187. doi: 10.3390/sports14050187 (PMC13210987; doi:10.3390/sports14050187)
Supplement: Supplementary file 1 [file sports-14-00187-s001.zip › Supplementary Table S4.pdf]

Supplementary Table S4. Characteristics of included studies (extended dataset).

Study	Population	Intervention	Dose / Duration	Primary Outcomes
de Hoyo et al., 2015	36 junior elite soccer, 17–19y	Eccentric-overload (flywheel)	10 weeks; 1–2 sessions/week; session duration not reported	Injury incidence/severity; CMJ; 10/20 m sprint
Lauersen et al., 2018	7,738 athletes, 12–40y	Strength training (various)	14–16 wks, 2–3×/wk; session duration not reported	Muscle injury incidence
Faude et al., 2017	704 youth, 10–19y (71% soccer)	Multimodal prevention (balance/strength/agility/power)	4–18 wks, 2–3×/wk; session duration not reported	Neuromuscular performance; skills
Grooms et al., 2013	41 male collegiate soccer, 18–25y	F-MARC 11+ warm-up	20 min, 5–6×/wk, 12 wks	LE injury risk; time lost
Emery & Meeuwisse, 2010	744 youth soccer, 13–18y	Soccer-specific NMT	15 min, ≥3×/wk, 20 wks	All/acute/LE/ankle/knee injury
Rudisill et al., 2022	Adult athletes	Eccentric, concentric, 11+, plyometric, stretching	not reported	Hamstring injury; strength; flexibility; fascicle length
Richmond et al., 2017	429 junior high, 11–16y	iSPRINT NMT warm-up	not reported	Sport/LE/time loss/knee injury
Mason et al., 2020	Team-sport athletes	Acute priming (resistance/cycling/running)	1–12 h pre, session duration not reported	Strength; power; sprint; jump
Steib et al., 2017	54–4,546 youth, 12–18y	NMT (strength/balance/plyometric/agility)	6–40 wks, 2–3×/wk, session duration not reported	LE injury incidence
Trecroci et al., 2020	9 subelite male soccer, 17–18y	Active recovery vs soccer-specific	1 session post-match session duration not reported	Sprint; repeated sprint; MVF
Booth et al., 2017	Rugby league players	Training load quantification	Not reported	Performance; injury characteristics
Nunes et al., 2024	17,260 athletes (all ages)	Nordic Hamstring Exercise	48 reps/wk (maint.), session duration not reported	Sprint; activation; eccentric strength; injury
Paravlic et al., 2024	275 adolescent male basketball, 15y	NMT warm-up	3 months, session duration not reported	Injury incidence; neuromuscular function
O'Malley et al., 2014	21,479 team sports	Multifaceted (balance/hamstring/eccentric)	not reported	Overall/LE/knee/ACL/ankle/hamstring injury
Vlachas & Paraskevopoulos, 2022	Footballers, 13–25y	FIFA 11+ warm-up	20 min, 3–5×/wk	Injury; strength; sprint; jump; balance

Boyd et al., 2023	427 pre-cond.; 261 control, 20–30y	Eccentric/isometric pre-conditioning	Single bout, 24 h pre, session duration not reported	CK; soreness; MVC; ROM
Li & Zhu, 2025	Adolescents, 12–18y, male	NMT	20–30 min, 1–2×/wk, ≥6 mo	LE; knee; ankle injury
Stojanović et al., 2022	112 basketball, 21.6y	Multicomponent NMT warm-up	~20 min, pre-training	Ankle/knee/non-contact LE injury
Bonato et al., 2018	160 elite female basketball	Bodyweight NMT	not reported	LE injury; strength; postural control
Bustos et al., 2020	31 soccer, 16–18y	Wearable resistance warm-up	8 wks, 2–3×/wk, session duration not reported	Sprint; jump; repeated sprint
Emery et al., 2019	1,067 junior high, 11–16y (54% female)	iSPRINT NMT warm-up	15 min/class, 12 wks	All/LE/medical/time loss/knee/ankle injury
Hilska et al., 2021	1,403 U11–U14 soccer (20% female)	NMT warm-up	20 min, 2–3×/wk, 20 wks	Acute LE; non-contact LE injury
Krutsch et al., 2019	529/601 elite male football	5-module prevention program	not reported	Severe knee injury
Bullock et al., 2025	154,561 (55% female)	NMT + PPE + policy/education	≥10 min, 2×/wk (NMT)	LE/ankle/ACL injury
Schache, 2012	942 male soccer	Eccentric hamstring (Nordic curl)	27×/10 wks, then 1×/wk, session duration not reported	Hamstring injury (all/new/recurrent)
Dolan et al., 2023	838 amateur rugby	Customized warm-up	Pre-training/match, season, session duration not reported	Acceptability; hamstring strain
Richmond et al., 2011	725/314 school youth, 11–15y	High-intensity NMT warm-up	15 min, 3×/wk, 12 wks	Sport injury; aerobic fitness; jump; balance
Rahlf & Zech, 2019	342 male soccer, 15.4y	NMT (11+) 10 vs 20 min	2×/wk, 6 month, session duration not reported	LE injury incidence
Emery et al., 2018	1,067 junior high (54% female)	iSPRINT NMT warm-up	15 min/class, 12 wks,	All; medical injury
Herzog et al., 2024	NFL players	Gradual 15-min ramp-up	Preseason, session duration not reported	LE strain incidence
Emery et al., 2020	1,067 school, 11–16y (54% female)	iSPRINT NMT warm-up	12 wks, session duration not reported	IRR; VO_2max; jump; balance
Berg et al., 2021	1,067 school, 11–16y (54% female)	iSPRINT NMT warm-up	12 wks, session duration not reported	IRR; balance; VO_2max; jump
Lopes et al., 2019	NR	Warm-up; NMT; eccentric	not reported	Muscle injury incidence
Behm et al., 2016	NR	Static/dynamic/PNF stretching	not reported	Performance; ROM; injury
Herman et al., 2012	~1,500, mostly female (13–26y)	NMT warm-up (various)	8–104 wks; 2–7×/wk, session duration not reported	LE; knee; ankle injury
Hübscher et al., 2010	Adolescents/young adults	Proprioceptive/NMT; balance	not reported	LE; knee; ankle injury
Emery et al., 2015	NR	NMT	not reported	LE; knee injury

Owen et al., 2013	26/23 elite male soccer	Structured prevention	2×/wk, season, session duration not reported	Muscle/total injuries
Bullock et al., 2010	Active populations	Overtraining; agility; PPE	not reported	Training-related injury

Abbreviations: ACL = Anterior Cruciate Ligament; CK = Creatine Kinase; IRR = Incidence Rate Ratio; LE = Lower Extremity; MVC = Maximal Voluntary Contraction; MVF = Maximal Voluntary Force; NHE = Nordic Hamstring Exercise; NMT = Neuromuscular Training; NFL = National Football League; PNF = Proprioceptive Neuromuscular Facilitation; PPE = Pre-Participation Examination; RCT = Randomized Controlled Trial; ROM = Range of Motion; RR = Relative Risk; VO_2max = Maximal Oxygen Uptake.
