# Supplementary material for: Exercise-Based Strategies from Warm-Up to Training: A Systematic Review of Performance Enhancement and Injury Prevention
Source: Sports (Basel). 2026 May 6;14(5):187. doi: 10.3390/sports14050187 (PMC13210987; doi:10.3390/sports14050187)
Supplement: Supplementary file 1 [file sports-14-00187-s001.zip › Supplementary Table S5.pdf]

Supplementary Table S5. Relative and incidence rate ratios for injury outcomes in controlled evaluations.

**Study (abridged)**	**Class**	**Metric**	**Comparator / notes**
Systematic review (NMT)	NMT/11+	RR = 0.61 (95% CI 0.49–0.77)	Favors intervention
FIFA 11+ meta-analysis	NMT/11+	RR ≈ 0.57	Favors intervention
School NMT (iSPRINT)	NMT/11+	IRR ≈ 0.54 overall; ≈ 0.36 LE	Favors intervention
High-intensity NMT (school)	NMT	IRR ≈ 0.29–0.30	Strong protective effect
Cluster RCT (basketball)	NMT	Ankle IRR = 0.26; Knee IRR = 0.32	Favors intervention
Elite men's football prevention modules	Multimodal	0.38 vs 0.68 / 1000 h	Fewer severe knees
Strength training meta-analysis	Strength	RR ≈ 0.34 (0.24–0.48)	Dose-dependent prevention
Eccentric hamstring (soccer)	Eccentric/NHE	NNT = 13 (any), 3 (recurrent)	Fewer hamstring injuries
Umbrella (NHE)	Eccentric/NHE	Qualitative synthesis	Supports eccentric strength + performance
Preseason ramp-up (NFL)	Ramp-up	~25% strain reduction	Progressive exposure

Direction clarified where original reporting used control/intervention as numerator; Abbreviations: RR = Relative Risk; IRR = Incidence Rate Ratio; CI = Confidence Interval; NMT = Neuromuscular Training; RCT = Randomized Controlled Trial; ACL = Anterior Cruciate Ligament; LE = Lower Extremity; NHE = Nordic Hamstring Exercise; NNT = Number Needed to Treat; NFL = National Football League; FIFA = Fédération Internationale de Football Association.
