# Supplementary material for: Exercise-Based Strategies from Warm-Up to Training: A Systematic Review of Performance Enhancement and Injury Prevention
Source: Sports (Basel). 2026 May 6;14(5):187. doi: 10.3390/sports14050187 (PMC13210987; doi:10.3390/sports14050187)
Supplement: Supplementary file 1 [file sports-14-00187-s001.zip › Supplementary Table S6.pdf]

Supplementary Table S6. Sprinting, jumping, and balance responses to targeted preparatory work.

Study (abridged)	Class	Outcomes (direction)
FIFA 11+ meta-analysis	NMT/11+	VJ +4.67 cm; 20 m sprint −0.38 s
Elite female basketball (cluster RCT)	NMT	CMJ +9.4%; Y-Balance +3–4%
Eccentric-overload (junior soccer)	Eccentric	CMJ ↑ (ES up to ~0.79); 10–20 m sprint ↑
School NMT (iSPRINT)	NMT	Dynamic balance ↑ (~+1.2 s)
Wearable resistance (soccer)	Warm-up/tool	Sprint ↓; SLJ ↑ (ES ~0.85–0.93)
High-intensity NMT (school youth)	NMT	VO_2max +2.14 ml/kg/min; VJ +4.16 cm
Stretching (review)	Stretching	Static −3.7%; Dynamic +1.3%; PNF −4.4% (task-dependent)

Abbreviations: NMT = Neuromuscular Training; RCT = Randomized Controlled Trial; VJ = Vertical Jump; CMJ = Countermovement Jump; SLJ = Standing Long Jump; VO_2max = Maximal Oxygen Uptake; PNF = Proprioceptive Neuromuscular Facilitation; ES = Effect Size.
