# Supplementary material for: Exercise-Based Strategies from Warm-Up to Training: A Systematic Review of Performance Enhancement and Injury Prevention
Source: Sports (Basel). 2026 May 6;14(5):187. doi: 10.3390/sports14050187 (PMC13210987; doi:10.3390/sports14050187)
Supplement: Supplementary file 1 [file sports-14-00187-s001.zip › Supplementary Table S7.pdf]

Supplementary Table S7. Full Search Strategy

Database	Search Date	Search Strategy (full string)	Filters / Limits	Notes
**PubMed**	August 2025	("neuromuscular training" OR "warm-up" OR "injury prevention" OR "eccentric training" OR "strength training" OR "FIFA 11+" OR "iSPRINT") AND ("muscle performance" OR "injury incidence" OR "athletic performance" OR "sprint" OR "jump" OR "balance") AND ("randomized controlled trial" OR "systematic review" OR "meta-analysis")	No date or language restrictions	MeSH terms and free-text terms combined
**Scopus**	August 2025	TITLE-ABS-KEY ("neuromuscular training" OR "warm-up" OR "injury prevention" OR "eccentric training" OR "strength training" OR "FIFA 11+" OR "iSPRINT") AND TITLE-ABS-KEY ("muscle performance" OR "injury incidence" OR "athletic performance" OR "sprint" OR "jump" OR "balance") AND TITLE-ABS-KEY ("randomized controlled trial" OR "systematic review" OR "meta-analysis")	No restrictions	Title, abstract, keywords
**Web of Science**	August 2025	TS=("neuromuscular training" OR "warm-up" OR "injury prevention" OR "eccentric training" OR "strength training" OR "FIFA 11+" OR "iSPRINT") AND TS=("muscle performance" OR "injury incidence" OR "athletic performance" OR "sprint" OR "jump" OR "balance") AND TS=("randomized controlled trial" OR "systematic review" OR "meta-analysis")	No restrictions	Topic search
**SPORTDiscus (EBSCO)**	August 2025	("neuromuscular training" OR "warm-up" OR "injury prevention" OR "eccentric training" OR "strength training" OR "FIFA 11+" OR "iSPRINT") AND ("muscle performance" OR "injury incidence" OR "athletic performance" OR "sprint" OR "jump" OR "balance") AND ("randomized controlled trial" OR "systematic review" OR "meta-analysis")	No restrictions	Field-based search
**EBSCO (general)**	August 2025	("neuromuscular training" OR "warm-up" OR "injury prevention" OR "eccentric training" OR "strength	No restrictions	Multi-database search

	training" OR "FIFA 11+" OR "iSPRINT") AND ("muscle performance" OR "injury incidence" OR "athletic performance" OR "sprint" OR "jump" OR "balance") AND ("randomized controlled trial" OR "systematic review" OR "meta-analysis")	

Search strategy development:

The search strategy was developed based on the research question and combined controlled vocabulary and free-text terms using Boolean operators.
